# Mathematical Analysis of Cytokine-Induced Differentiation of Granulocyte-Monocyte Progenitor Cells

**DOI:** 10.3389/fimmu.2018.02048

**Published:** 2018-09-18

**Authors:** Bronson R. Weston, Liwu Li, John J. Tyson

**Affiliations:** ^1^Program in Genetics, Bioinformatics, and Computational Biology, Virginia Polytechnic Institute and State University, Blacksburg, VA, United States; ^2^Department of Biological Sciences, Virginia Polytechnic Institute and State University, Blacksburg, VA, United States

**Keywords:** granulopoiesis, monopoiesis, differentiation, cytokines, mathematical modeling, temporal dynamics, monocytic myeloid-derived suppressor cells

## Abstract

Granulocyte-monocyte progenitor (GMP) cells play a vital role in the immune system by maturing into a variety of white blood cells, including neutrophils and macrophages, depending on exposure to cytokines such as various types of colony stimulating factors (CSF). Granulocyte-CSF (G-CSF) induces granulopoiesis and macrophage-CSF (M-CSF) induces monopoiesis, while granulocyte/macrophage-CSF (GM-CSF) favors monocytic and granulocytic differentiation at low and high concentrations, respectively. Although these differentiation pathways are well documented, the mechanisms behind the diverse behavioral responses of GMP cells to CSFs are not well understood. In this paper, we propose a mechanism of interacting CSF-receptors and transcription factors that control GMP differentiation, convert the mechanism into a set of differential equations, and explore the properties of this mathematical model using dynamical systems theory. Our model reproduces numerous experimental observations of GMP cell differentiation in response to varying dosages of G-CSF, M-CSF, and GM-CSF. In particular, we are able to reproduce the concentration-dependent behavior of GM-CSF induced differentiation, and propose a mechanism driving this behavior. In addition, we explore the differentiation of a fourth phenotype, monocytic myeloid-derived suppressor cells (M-MDSC), showing how they might fit into the classical pathways of GMP differentiation and how progenitor cells can be primed for M-MDSC differentiation. Finally, we use the model to make novel predictions that can be explored by future experimental studies.

## Introduction

Hematopoietic stem cells differentiate into blood cells (neutrophils, monocytes, red blood cells, etc.) in a finely regulated process called hematopoiesis. In this branching process, each branch point represents a cell differentiating into one of two alternative lineages. Stimulatory factors, such as cytokines, induce differentiation into one lineage over another, and cross-antagonistic transcription factors maintain commitment to the chosen lineage (1, 2). In the myeloid branch of hematopoiesis, granulocyte-monocyte progenitor (GMP) cells differentiate into essential cells of the innate immune system, including granulocytes (neutrophils, eosinophils, and basophils) and monocytes (which further differentiate into macrophages and dendritic cells), depending on the local concentrations of specific colony stimulating factors (CSFs) (3, 4). Therefore, proper orchestration of GMP differentiation is of vital significance to human health. For instance, myeloid cells are often targeted with CSFs to treat a variety of diseases including arthritis, infections, pneumonia, cancer, type 1 diabetes, and neutropenia (5–7). A better understanding of the biological responses of myeloid cells to these stimuli will be useful to refine and develop new therapeutic strategies.

**Figure 1:**
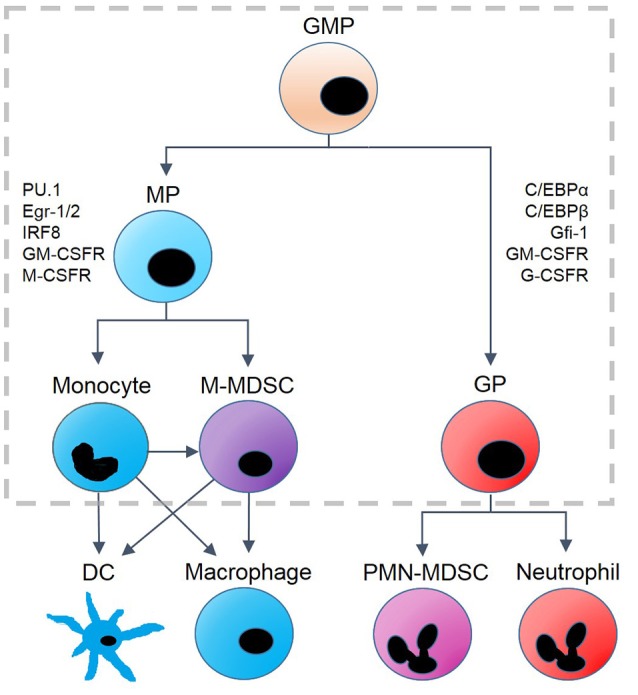
Hematopoietic lineages derived from granulocyte-monocyte progenitor (GMP) cells. GMP differentiation into monocyte progenitors (MP) or granulocyte progenitors (GP) results in changes of protein expression. GP cells are associated with the upregulation of C/EBPα, C/EBPβ, Gfi-1, G-CSFR and GM-CSFR. Monopoiesis is associated with upregulation of PU.1, Egr-1/2, IRF8, M-CSF, and GM-CSFR. MP cells differentiate into monocytes and monocytic myeloid-derived suppressor cells (M-MDSC), and monocytes can be converted into M-MDSCs under some conditions. Monocytic precursors terminally differentiate into dendritic cells (DC) and macrophages, while GP cells differentiate into polymorphonuclear (PMN-) MDSCs and neutrophils as well as eosinophils and basophils (not shown). The model we propose is designed to capture the dynamics within the gray, dashed box.

It is well known that G-CSF and M-CSF induce differentiation of granulocytes and monocytes, respectively, while a range of concentration-dependent behaviors can be observed in response to GM-CSF. GMP populations favor monopoiesis at low concentrations of GM-CSF and granulopoiesis at higher concentrations (1, 8–10). Figure 1 provides a schematic illustration of GMP differentiation. Critical proteins for granulocytic commitment include CCAAT enhancer-binding proteins (C/EBPα and C/EBPβ), growth-factor independent-1 protein (Gfi-1), GM-CSF receptor (GM-CSFR), and G-CSF receptor (G-CSFR). Proteins involved in monocytic commitment include PU.1 (a protein encoded by the *SPI1* gene in humans), early growth response proteins 1 and 2 (Egr-1 and Egr-2), interferon-regulatory factor 8 (IRF8), M-CSF receptor (M-CSFR), and GM-CSFR (1, 11–17).

Despite the vital roles that cells of the GMP lineage play in the body, much is still unknown about the dynamics of their differentiation. Laslo et al. suggested that PU.1 and C/EBPα stimulate cross-antagonistic transcription factors, Egr-2 and Gfi-1, to maintain granulocytic and monocytic commitment, respectively (15). This cross-antagonistic relationship, which is thought to be critical to gene regulation within the myeloid lineage, was modeled by Laslo et al. with a simple, symmetrical, interaction motif that exhibits lineage commitment of monocytes and granulocytes in response to external signals. However, the simple motif they propose cannot explain more complex behavior, such as GMP responses to low and high doses of GM-CSF. It is also not well understood how GMP cells respond to varying concentrations and combinations of cytokines, nor how GMP cells differentiate into myeloid-derived suppressor cells (MDSCs), which are immature myeloid cells that exhibit both granulocytic and monocytic traits (18–20). MDSCs have anti-inflammatory properties and serve a beneficial role in a variety of pathological conditions (21, 22) nonetheless, they are more often associated with promotion of cancer growth. It is well documented that MDSCs promote angiogenesis and metastasis, and many studies suggest that suppression of these cells may be a promising clinical target in cancer therapy (18, 23–28). While originally lumped into one heterogeneous group, MDSCs have been reclassified into two separate types: polymorphonuclear (PMN)-MDSCs and monocytic (M)-MDSCs (18, 23, 29). Distinguishing between these subsets is crucial, as they have different mechanisms of immunosuppression, respond to different cytokines, and are more closely associated with different tissues and cancers (23, 30, 31). While PMN-MDSCs typically exist at higher population densities than M-MDSCs, M-MDSCs are more potent suppressors of inflammation on a per-cell basis (30, 32). Of the two subsets, we will focus on M-MDSCs, as our model does not include the downstream transcription factors necessary to distinguish between PMN-MDSCs and other cells of the granulocyte lineage.

In this paper, we propose a new model of the internal regulatory network that governs GMP cell differentiation and how various cytokine signals feed into this regulatory network. We convert our network diagram into a set of nonlinear ordinary differential equations (ODEs) and study their properties by dynamical systems theory. We first explore the polarization of GMP cells resulting from G-CSF and M-CSF signals. Next we explore the dynamics of the system in response to GM-CSF and propose a mechanism driving the complex behavior observed in GM-CSF experiments. We also explore how M-MDSCs may fit into this differentiation scheme, including the stability of the state and the nature of the phenotype itself. Finally, we evaluate the system's response to cytokine combinations and provide insight into the spectrum of behaviors induced by signaling crosstalk.

## Materials and methods

### The proposed regulatory network and its molecular basis

**Figure 2:**
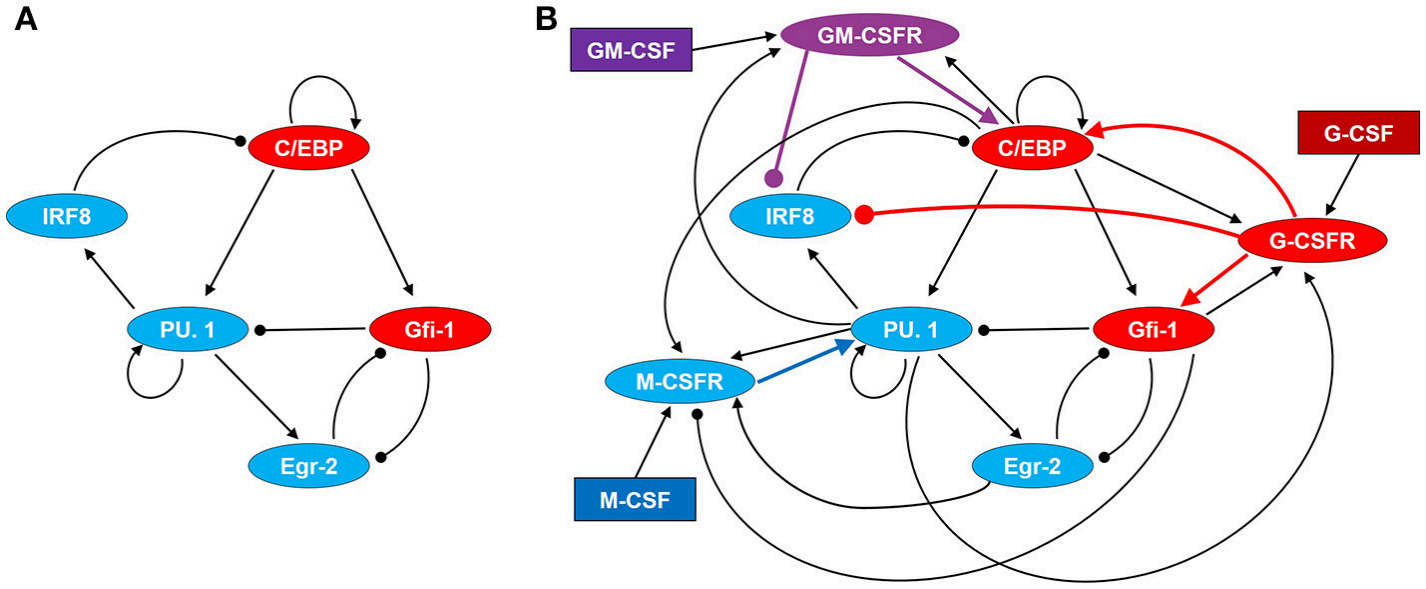
Regulatory network driving GMP differentiation in response to M-CSF, G-CSF, and GM-CSF. **(A)** Transcription factor network. **(B)** Cytokine signaling and regulatory network. Regulatory motifs are expressed in terms of direct and indirect interactions among proteins, where a line with an arrow head represents the activation of one protein by another and a line with a circular head represents inhibition. Blue and red ovals denote proteins highly expressed in monocyte and granulocyte lineages, respectively. GM-CSFR is represented by a purple oval as it can signal for both monopoiesis and granulopoiesis. Cytokines are denoted by rectangles.

PU.1 and C/EBPα are thought to be master regulators of myelopoiesis, as C/EBPα favors granulopoiesis and PU.1 favors monopoiesis (33, 34). In this subsection we summarize the experimental evidences characterizing the interactions of PU.1, C/EBPα and their closely interacting partners, in order to motivate the regulatory network (Figure 2A) that we will use to understand the differentiation of GMP cells.

First of all, we note that the roles of C/EBPα and C/EBPβ appear to be redundant in hematopoiesis. When the *C/EBP*β gene was knocked in to the *C/EBP*α locus, no significant changes in hematopoiesis or gene expression were ascertained. Since these proteins have highly conserved C-terminal dimerization- and DNA-binding domains, it is reasonable to assume that they bind to the same promoter sites (35). It is possible, indeed probable, that these proteins have differences in regulation at the transcriptional level; however, it has been demonstrated that GM-CSF and G-CSF upregulate both C/EBPα and C/EBPβ (36, 37). Furthermore, both C/EBPα and C/EBPβ exhibit positive autoregulation (38, 39). Due to this overlap of structure and function, we lump C/EBPα and C/EBPβ into one node/variable, called C/EBP. Unless otherwise specified, “C/EBP” will refer to the combination of C/EBPα and C/EBPβ rather than the entire family of C/EBP transcription factors.

The interactions between PU.1 and C/EBP are intriguing, as they have both antagonistic and synergetic relationships (Figure 2A). It has been demonstrated that the promoter of the *SPI1* gene (encoding the PU.1 protein) has multiple potential C/EBP binding sites, and that C/EBPα can induce PU.1 expression by directly binding to the promoter to activate transcription (40, 41). Alternatively, C/EBPα can inhibit PU.1 indirectly by upregulating the *Gfi1* gene (42). Gfi-1 in turn, physically binds with PU.1 to inhibit its activity as a transcription factor (43). These affects are amplified, since PU.1 auto-activates its own promoter site (44). Furthermore, Gfi-1 binds directly to numerous PU.1 target genes to repress PU.1's transcriptional activities (43). We suspect that this process could further inhibit *SPI1* transcription, given possible positive feedback loops between PU.1 and its downstream targets. In addition, PU.1 antagonizes C/EBP either directly or through activation of IRF8, which creates a mutual inhibition circuit between PU.1 and C/EBP (45, 46). IRF8 physically interacts with C/EBPα to prevent it from binding to chromatin and promoting transcription of target genes (47). While, to the best of our knowledge, no studies have demonstrated that C/EBPβ is inhibited by IRF8, it has been demonstrated that IRF8 also binds to and inhibits C/EBPε, suggesting that it may function similarly on C/EBPβ (47). Furthermore, it has been shown that IRF8-knockdown induces C/EBPβ expression in dendritic cells (48). PU.1 has also been shown to inhibit the transcriptional activity of C/EBPα and C/EBPβ in adipocyte differentiation via direct protein-protein interactions (46). Similar interactions may occur in myelopoiesis, as it has been shown that C/EBPα directly interacts with PU.1 to block PU.1-induced dendritic cell commitment (49). Despite this evidence, we do not model the potential direct interaction between PU.1 and C/EBP as regulatory details are not clear in regard to GMP differentiation, and a mutual inhibitory relationship is already captured within our motif.

Egr-2, another downstream transcription factor promoted by PU.1, forms a complex with Nab-2 to inhibit Gfi-1. Similarly, Gfi-1 regulates Egr-1 and-2 to reduce the concentration of the Nab/Egr complex (15, 50). Thus, the Egr-Gfi-1 relationship creates a second layer of antagonism within this myeloid differentiation system. Since Gfi-1 can inhibit Egr expression, but not Nab, we simplify our model by excluding Nab, with the assumption that the concentrations of the Egr-Nab complex will be proportional to the concentration of Egr.

Within our model, three receptors (M-CSFR, G-CSFR, and GM-CSFR) transduce cytokine signals to regulate transcription factor activity (Figure 2B). These transcription factors, in turn, regulate expression of the receptors, thereby creating positive and negative feedback loops. We model PU.1 as the primary target of M-CSF signaling, since M-CSF induces monocyte differentiation and PU.1 is a master regulator of monopoiesis. Although we do not know of any confirmed pathway, it is known that M-CSF can signal through ERK to activate a transcription factor, Sp1, which can bind to multiple sites on the *SPI1* promoter (44, 51). Thus, it is plausible that M-CSF induces PU.1 expression through Sp1. PU.1, as well as C/EBPα, C/EBPβ, and Egr-2, bind to the M-CSFR promoter region to activate transcription, creating a positive feedback loop between PU.1 and M-CSFR (1, 13, 50, 52). Gfi-1, however, binds to the promoter to disrupt transcription (53).

G-CSFR signals primarily to STAT3 and SHP2, resulting in upregulation of C/EBPα and C/EBPβ (33, 36, 54). SHP2 activates RUNX1, which in turn promotes Gfi-1 expression via promoter regulation (55, 56). Thus, G-CSFR activates Gfi-1 by a mechanism independent of C/EBPα, and we simplify this interaction in our model by having G-CSFR directly upregulate Gfi-1 when bound to its ligand. C/EBP, PU.1 and Gfi-1 promote G-CSFR activity, but through different mechanisms. C/EBPα, C/EBPβ, and PU.1 bind directly to the G-CSFR promoter to upregulate G-CSFR expression, while Gfi-1 represses miR-21 and miR196b, which both inhibit G-CSF-mediated granulopoiesis (13, 57). Furthermore, Gfi-1 promotes Ras guanine nucleotide releasing protein 1 (RasGRP1), which regulates G-CSFR-induced Ras activation (58). C/EBPα transactivation activity is also enhanced by G-CSF-induced Ras signaling (59). Thus, while C/EBP and PU.1 directly induce G-CSFR expression, Gfi-1 enhances the capacity for G-CSFR signaling by regulating signal transduction intermediates. These interactions create a positive feedback loop consisting of C/EBP, Gfi-1, and G-CSFR. This feedback loop extends when considering the effects of G-CSFR signaling on IRF8, as G-CSF can inhibit IRF8 indirectly through the STAT3 pathway and SHP2. It was demonstrated that SHP2 activates STAT5 which inhibits transcription of the *IRF8* gene (60, 61).

GM-CSF drives granulopoiesis through similar mechanisms as G-CSF; however, GM-CSF signals primarily through STAT5 rather than STAT3 (37, 62). Experimental evidence suggests that upregulation of C/EBPα in pre-basophil and mast cell progenitors is STAT5-dependent (63). Thus, it is likely that GM-CSF-activated STAT5 results in the upregulation of C/EBP in GMP cells. As mentioned earlier, STAT5 also represses IRF8 transcription, and therefore GM-CSFR signaling should repress IRF8. GM-CSFR expression is upregulated by C/EBPα, C/EBPβ and PU.1 via transcriptional promotion (1, 13, 64).

### Conversion of the interaction diagram into a mathematical model

To convert the interaction diagram in Figure 2B into a set of nonlinear ODEs, we use a formalism called “standard component” modeling (65). Each of the eight proteins in Figure 2B (excluding cytokines) is governed by an ODE of the form:

(1) $$\begin{array}{l} \frac{dX_{i}}{dt}=\rho_{i}\left(\frac{1}{1+e^{-\sigma_{i}W_{i}}}-X_{i}\right) \end{array}$$

(2) $$\begin{array}{l} W_{i}=\omega_{i}^{o}+\;\sum_{j\;=\;1}^{N}\omega_{i,j}X_{j} \end{array}$$

The (relative) concentration or activity of protein *i* is denoted by the variable *X*_*i*_(*t*), 0 ≤ *X*_*i*_(*t*) ≤ 1. The function W_*i*_(*X*_*j*_) accounts for all interactions within the network that directly affect the rate of change of *X*_*i*_ such that ω_*i, j*_ quantifies the direction and strength of the affect that protein *j* exerts on protein *i*. Negative values represent inhibition while positive values represent activation. The time scale for the rate of change of *X*_*i*_(*t*) is determined by 1/ρ_*i*_. The value of $\omega_{i}^{o}$ determines the value of *X*_*i*_ when it is not receiving stimulus from any *X*_*j*_. One unit of the time variable, *t*, is roughly 2 h in our simulations.

The nonlinear function, $H(W)=1/(1+e^{-\sigma \;W})$, in this ODE is a sigmoidal function of *W*, with steepness determined by the parameter σ. Many biological phenomena such as phosphorylation cascades and transcriptional regulation are characterized by sigmoidal response curves. Our sigmoidal function *H*(*W*) captures such behavior in a very convenient way. As an example, we show the case of C/EBP activity:

(3) $$\frac{\text{d}{\left[\text{C/EBP}\right]}_{T}}{\text{dt}}=\rho_{\text{TF}}\left(\frac{\text{1}}{{\text{1+e}}^{-\sigma {\text{W}}_{\text{C}/\text{EBP}}}}-{\left[\text{C}/\text{EBP}\right]}_{T}\right)$$

(4) $$\begin{array}{l} W_{\text{C}/\text{EBP}}=\omega_{\text{C/EBP}}^{o}+\;\omega_{\text{C/EBP},\text{C/EBP}}{\left[\text{C}/\text{EBP}\right]}_{\text{F}}\\ \;+\omega_{\text{C}/\text{EBP},\text{GMCSFR}}\left[\text{GMCSFR }:\text{ GMCSF}\right]\\ \;+\;\omega_{\text{C}/\text{EBP},\text{GCSFR}}\left[\text{GCSFR }:\text{ GCSF}\right] \end{array}$$

We use $\rho_{TF}$ rather than $\rho_{C/EBP}$ as all transcription factors have the same time scale in our model.

Note that this ODE distinguishes between two concentrations of C/EBP: its “total” concentration, [C/EBP]_T_, and the concentration of the “free” form of the protein, [C/EBP]_F_. C/EBP is considered free when it's not bound to IRF8, therefore [C/EBP]_F_ represents the active portion of [C/EBP]_T_, where

(5) $${\left[\text{C}/\text{EBP}\right]}_{\text{T}}=\;{\left[\text{C}/\text{EBP}\right]}_{\text{F}}+[\text{C}/\text{EBP }:\text{ IRF8}]$$

and [C/EBP:IRF8] denotes the concentration of the C/EBP-IRF8 complex. Similarly,

(6) $${\left[\text{IRF8}\right]}_{\text{T}}=\;{\left[\text{IRF8}\right]}_{\text{F}}+\;[\text{C}/\text{EBP }:\text{ IRF8}]$$

Since protein-protein binding is governed by the law of mass action, and the timescale for association and dissociation of proteins is likely to be much faster than other time scales in the model, we assume that, at any given time, the reaction [C/EBP]_F_ + [IRF8]_F_ ⇋ [C/EBP:IRF8] is at equilibrium. Thus,

(7) $$K_{\text{eq}}=\frac{\left[\text{C}/\text{EBP }:\text{ IRF8}\right]}{{\left[\text{C}/\text{EBP}\right]}_{\text{F}}\;\cdot \;{\left[\text{IRF8}\right]}_{\text{F}}}$$

Using Equations (5–7) we can obtain an equation for [C/EBP]_T_ as a function of [C/EBP]_F_ and [IRF8]_T_:

(8) $${\left[\text{C}/\text{EBP}\right]}_{\text{T}}={\left[\text{C}/\text{EBP}\right]}_{\text{F}}\cdot \;\left(1\;+\frac{K_{eq}\cdot {\left[\text{IRF8}\right]}_{\text{T}}}{1+K_{\text{eq}}\cdot {\left[\text{C}/\text{EBP}\right]}_{\text{F}}}\;\right)$$

and an equation for [C/EBP]_F_ as a function of [C/EBP]_T_ and [IRF8]_T_

(9) $${\left[\text{C}/\text{EBP}\right]}_{\text{F}}=\frac{1}{2}\left(-b+\sqrt{b^{2}+\frac{\text{4}{\left[\text{C}/\text{EBP}\right]}_{\text{T}}}{K_{\text{eq}}}}\;\right)$$

(10) $$\text{where }b={\left[\text{IRF}8\right]}_{\text{T}}+\frac{1}{K_{eq}}-{\left[\text{C/EBP}\right]}_{\text{T}}.$$

Regarding binding of external cytokines to their membrane-bound receptors, we assume that the cytokine concentration, [L], is constant and much greater than the total concentration of receptors, [R]_T_. In this case, the concentration of the receptor:cytokine complex, [R:L], is given by the function:

(11) $$[\text{R }:\text{ L}]=\frac{\left[\text{L}\right]{\left[\text{R}\right]}_{\text{T}}}{\left[\text{L}\right]+K_{d}}$$

where *K*_d_ is the dissociation constant of the receptor:cytokine complex. The cytokine concentrations are “inputs” to the model, the total receptor concentrations are dynamic variables of the model.

Parameter values were hand-tuned so that the behavior of the system in response to cytokines aligns with experimental observations. For a more detailed discussion on parameter tuning, a table of parameter values and the complete set of equations constituting our mathematical model, see the Supplementary Material.

### Computational methods

All quantitative simulations were computed using the deterministic ODE solver, ode45, in MATLAB. To simulate a population of GMP cells, we generate a set of cells with stochastically varying initial conditions, taking the steady state concentrations of all variables in a naïve GMP cell (with no cytokine stimulation) and varying each initial concentration by a random factor drawn from a normal distribution with mean = 1 and standard deviation = 0.2.

Although our model consists of eight nonlinear ODEs, we characterize its behavior in a pseudo-phase plane spanned by only two variables: [PU.1] and [C/EBP]_T_. To do so, we demand that the other six variables are in pseudo-steady state by setting the right-hand-sides of those six ODEs = 0. Looking at the structure of those six ODEs (in the Supplementary Material), we see all six pseudo-steady state concentrations can be written as explicit functions of [PU.1], [C/EBP]_F_ and [Gfi1]. For example, the pseudo-steady state value of [IRF8]_T_ is a function of [PU.1] and the complexes [GMCSFR:GMCSF] and [GCSFR:GCSF]. From Equation (11), these concentrations are functions of the stimulus parameters, [GMCSF] and [GCSF], and of the pseudo-steady state values of [GMCSFR]_T_ and [GCSFR]_T_, which in turn are functions, respectively, of [PU.1] and [C/EBP]_F_ and of [PU.1], [C/EBP]_F_, and [Gfi1]. From Equation (7), we can express [C/EBP]_T_ as a function of [C/EBP]_F_ and [IRF8]_T_. By this line of reasoning, we can reduce the 8-dimensional system of ODEs to three variables, [PU.1], [C/EBP]_T_, and [Gfi1].

To reduce this system to two variables, we must express the pseudo-steady state concentration of Gfi-1 as a function of [PU.1] and [C/EBP]_T_. We cannot provide an explicit representation of this function but we can find it by an iterative numerical approach. For any combination of values of [C/EBP]_F_ and [PU.1], we subdivide the interval [Gfi1] ∈ [0, 1] into 100 subintervals of length 0.01 and compute the value of *d*[Gfi1]/*dt* at the ends of each segment (see, e.g., Figure S1). Any subinterval for which the sign of *d*[Gfi1]/*dt* changes is further subdivided into ten sub-subintervals, and the iterative process is repeated until we have good approximation of the pseudo-steady state (pss) value(s) of [Gfi1] for the given pair of [PU.1] and [C/EBP]_F_ values. We solve for [Gfi1]_pss_ with a tolerance of 10^−9^ (We acknowledge that Newton's method would have been a more efficient approach; however, this method works all the same).

Since each point along the PU.1 pseudo-nullcline has a fixed value of [C/EBP]_F_, we calculate solutions for the PU.1 nullcline over a range of [C/EBP]_F_ values. Stringing these solutions together, we acquire the desired nullcline. We can find the [PU.1] nullcline solutions for any [C/EBP]_F_ value, as we did for finding [Gfi1]_pss_, by generating a subdivision of [PU.1] values between 0 and 1. For each [PU.1] value we solve for [Gfi1]_pss_ as above. As mentioned previously, given a set of [C/EBP]_F_, [PU.1], and [Gfi1] concentrations we can numerically solve for every other variable's steady state solution. Thus, we are able to calculate *d*[PU.1]/*dt*, and then to find the nullcline for [PU.1], where *d*[PU.1]/*dt* = 0. In this case, we use a tolerance of 10^−7^ rather than 10^−9^. Similarly, we solve for the [C/EBP]_F_ nullcline using the same method with a tolerance of 10^−8^. (We use different tolerances for different solutions due to different sensitivities and computational times.) Once we have acquired our pseudo-nullclines for the [C/EBP]_F_–[PU.1] phase plane, we convert [C/EBP]_F_ to [C/EBP]_T_ and plot the nullclines in the [C/EBP]_T_–[PU.1] phase plane. While unconventional and computationally taxing, our methods yield smoother, more accurate curves than we were able to obtain using XPPAUT, and we could do all our computations within MATLAB rather than switching between MATLAB and some other bifurcation software.

Bifurcation diagrams were generated in a similar, non-conventional fashion. Bifurcation diagrams are based on steady state solutions of all 8 variables. Given a set of approximate steady state values, we use MATLAB's fminsearch function to find values of [C/EBP]_F_ and [PU.1] that minimize the objective function ((*d*[C/EBP]_F_/*dt*)^2^ + (*d*[PU.1]/*dt*)^2^)^1/2^ given that all other variables are at steady state (To find [Gfi-1]_ss_ we use the same iterative technique specified earlier). Once a solution for [C/EBP]_F_ and [PU.1] is found, we use that solution as an initial guess for the next step (after making a small change to the value of the bifurcation parameter) and search for the new solution. Close to saddle-node bifurcation points, where the derivative of [PU.1]_ss_ with respect to the bifurcation parameter becomes very large, we hold the bifurcation parameter constant, and take a small step in the value of [PU.1]. We then use fminsearch to find the solution of [C/EBP]_F_ and the bifurcation parameter at that given [PU.1] value. To evaluate stability, we use a typical eigenvalue analysis.

To construct heat maps, we simulate 500 stochastically generated cells, using the method specified earlier, under each cytokine condition. Using the differentiated population's ratios, each pixel was assigned an RGB value determined by the following equation:

(12) $$\begin{array}{l} \left[\text{R},\text{G},\text{B}\right]=\left[{\left(\frac{\text{GP}}{\phi }\right)}^{\frac{1}{3}},\;{\left(\frac{\text{MMDSC}}{\phi }\right)}^{\frac{1}{3}},\;{\left(\frac{\text{MO}}{\phi }\right)}^{\frac{1}{3}}\right] \end{array}$$

(13) $$\begin{array}{l} \phi =Max\left(\text{GP},\text{ MMDSC},\text{ MO},\text{ GMP}\right) \end{array}$$

where the red, green and blue intensities are a function of the fraction of the population which differentiated into granulocytes progenitors (GP), M-MDSCs, and monocytes (MO), respectively, over the size of the largest population category (including undifferentiated GMP cells).

For those interested in exploring our model further, we provide two resources for utilizing our model and conducting simulations of your own. The supplementary code provides an ODE file, a stochastic simulation function and a user friendly MATLAB script, “MainScript.m,” to produce time course simulations and figures as well as stochastic simulations under user specified conditions. A more extensive resource is provided online at https://github.com/bronsonweston/GMP-Modeling, which includes all algorithms previously mentioned and provides a script, “FigureGenerating.m,” to easily reproduce any of our results. This code can also be used as an example script to conduct alternative simulations not explored in this study.

## Assumptions

As with any model, we have made several simplifying assumptions to avoid unnecessary complexity. First, we ignore autocrine feedback loops of the GMP lineage. We maintain constant cytokine concentration(s) in order to evaluate the effects of the stimulus input, rather than accounting for how the cell may change external conditions. We assume that the cytokine production of an individual cell has a negligible impact on the initial decision-making process of GMP differentiation. Additionally, we assume that all protein isoforms function similarly in the context of our network. For example G-CSFR has seven isoforms, four of which are involved in granulopoiesis (66). We assume that [G-CSFR] is the sum of these isoforms, weighted according to the contribution of each to granulopoiesis.

The GMP differentiation network has many mechanisms for generating sigmoidal nonlinearities, such as dimerization of receptor subunits and cooperativity of transcription factor binding to DNA promoter sites. We assume that our sigmoidal functions, *H*(*W*_*i*_) = 1/(1+*e*^−σ*Wi*^), adequately capture the cumulative non-linear effects of these molecular mechanisms.

In addition, we assume that all transcription factors function at the same time scale, and all receptors function at the same, ten-fold slower time scale (ρ_R_ = ρ_TF_/10). It is hard to know for sure what timescales these proteins are functioning on. While transcription factors are often functional immediately after synthesis, receptors must be trafficked to the periphery of the cell, diffuse within the cell membrane, assemble with other subunits, and bind to cytokines before a signal can be transduced back into the cell, after which the signal itself may take some time to get to its downstream target. At the very least, we would expect a significant time delay between production of the receptor and its impact on the expression of down-stream genes. For these reasons, we justify using a slower timescale for receptors than for transcription factors.

Finally, we assume that receptor activation does not have a significant negative feedback mechanism. Although it has been observed that the level of a receptor, such as M-CSFR and GM-CSFR, is reduced after stimulation by its own ligand (67), we choose to ignore these feedback loops, as we are interested in the initial aspects of cell differentiation, which are dominated by the positive feedback loops included in our model.

## Results

### A motif for GMP cell differentiation

The primary objective of this paper is to construct and analyze a dynamic model of the differentiation of GMP cells into monocyte and granulocyte lineages. Before describing the results derived from our model, we compare it briefly to the work of Laslo et al. who proposed a simple, symmetric model of the interactions among C/EBPα, PU.1, Gfi-1, and Egr (15). The purpose of their model was to demonstrate that mutual antagonism between Gfi-1 and Egr can be a mechanism for inducing commitment of the monocytic and granulocytic lineages. While achieving its intended purpose, the model's forced symmetry and its neglect of critical regulatory mechanisms limit its predictive capacity and its ability to explain more complex phenomena of myelopoiesis. We improve upon the Laslo model with new, biologically relevant interactions, including a fifth transcription factor, IRF8, as well as new signaling pathways, CSF receptors, and regulatory mechanisms for these receptors. These additional interactions break the symmetry of Laslo's model but extend the range of behaviors we can model. Rather than modifying the equations of Laslo's model, we derive a new set of equations based on our standard-component modeling approach. Justification for these changes can be found in the methods section.

**Figure 3:**
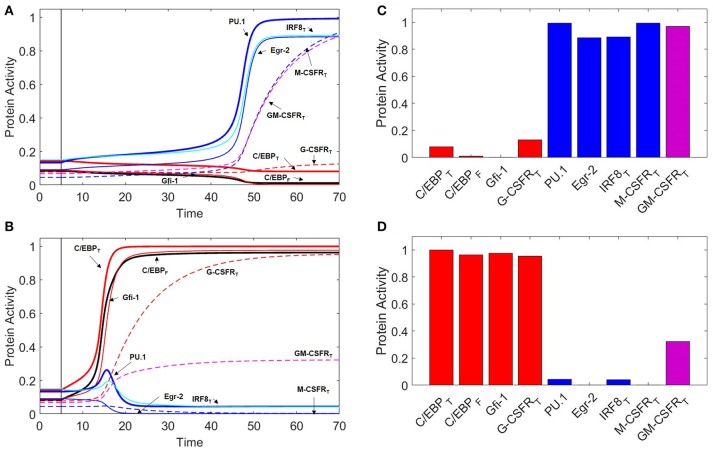
Protein activity over time during M-CSF and G-CSF induced differentiation. For *t* ≤ 5, there is no cytokine stimulation. When *t* > 5, [MCSF] = 1 in **(A)**, and [GCSF] = 1 in **(B)**. Under M-CSF stimulation, the GMP cell differentiates into a monocyte (PU.1^high^, C/EBP_low_) while under G-CSF stimulation the cell differentiates into a granulocyte progenitor (PU.1_low_, C/EBP^high^). Thick red line = [C/EBP]_T_, black line = [C/EBP]_F_, thick blue line = [PU.1], thin red line = [Gfi-1], thin blue line = [Egr-2], cyan line = [IRF8]_T_, dashed red line = [G-CSFR]_T_, dashed blue line = [M-CSFR]_T_, dashed magenta line = [GM-CSFR]_T_. One time unit = 2 h. **(C)** Final protein activity levels for simulation A indicate that M-CSF induces monopoiesis. **(D)** Final protein activity levels for simulation B indicate that G-CSF induces granulopoiesis.

Our motif for GMP cell differentiation is depicted in Figure 2B. We convert this signaling network into a set of non-linear ODEs (see Table S1) with parameter values specified in Table S2. Sample simulations for monocyte and granulocyte differentiation are presented in Figure 3. To gain some insight into these simulations, we use the notion of a “phase plane” from dynamical systems theory (68–70). Although our system of eight nonlinear ODEs defines an eight-dimensional phase space, we can reduce it to a two-dimensional phase plane by making pseudo-steady state approximations on six of the eight variables, leaving the concentrations of “master regulators” PU.1 and C/EBP as the fundamental state variables. The method by which we affect this reduction is explained in the section on “computational methods.”

**Figure 4:**
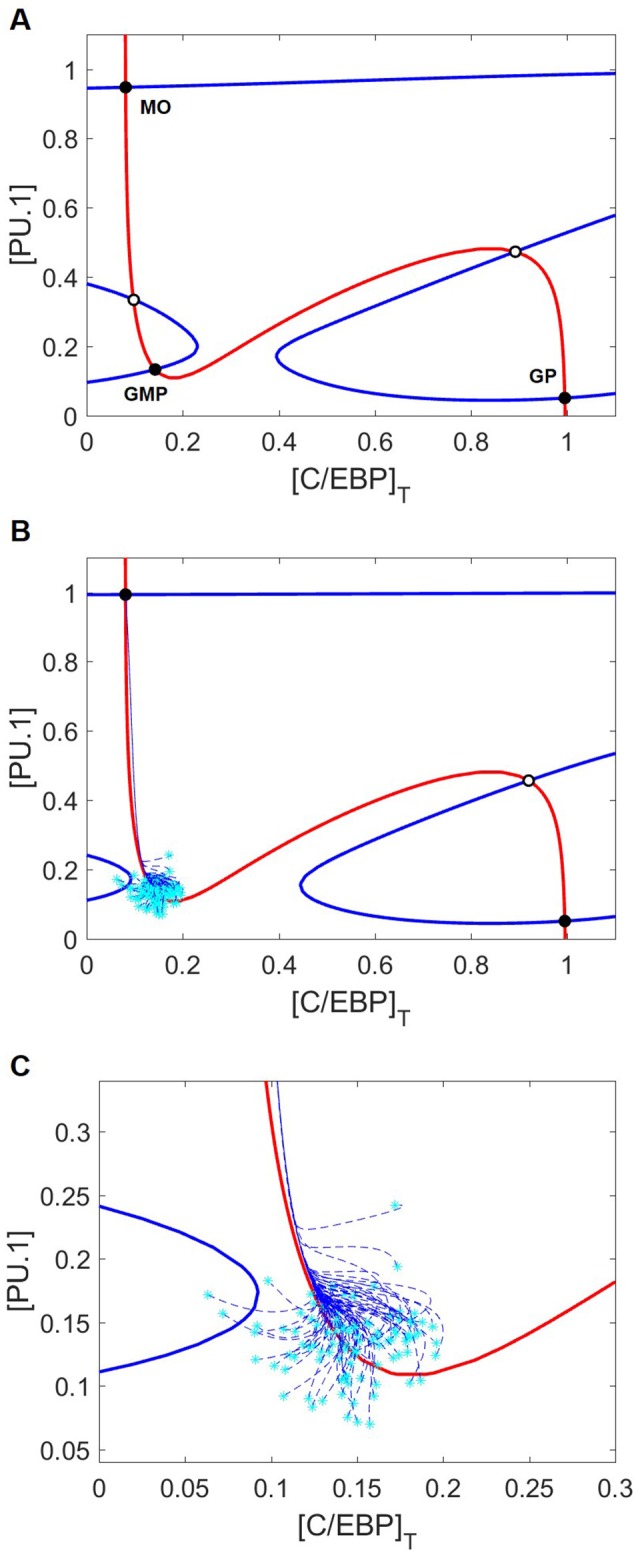
Nullcline movement due to M-CSF eliminates the GMP state and induces differentiation into the monocyte lineage. Blue and red lines are the PU.1 and C/EBP nullclines, respectively. Black circles and white circles designate stable and unstable steady states, respectively. Cyan asterisks represent stochastically generated initial conditions, while blue dashed lines represent the cellular trajectories of monopoiesis from these initial conditions. **(A)** Nullclines with no cytokine stimulus. Of the five intersection points, three represent stable steady states: MO, GMP, and GP mark the monocyte, naïve GMP and granulocyte progenitor (GP) states, respectively. Without cytokine stimulation, GMP cells will remain in the stable naïve state, even though monocyte and granulocyte progenitor cell states are also stable nodes of attraction. **(B)** Nullclines with M-CSF stimulation [(MCSF) = 1]. Only the PU.1 nullcline moves in response to M-CSF, resulting in loss of the GMP state. Cells starting in the neighborhood of the GMP state that has disappeared find themselves in the domain of attraction of the stable monocyte-progenitor cell state. **(C)** A closer look at the initial trajectories of stochastically generated cells reveals that they converge along the same path toward the monocyte state despite different initial conditions.

On the phase plane (Figure 4A), we plot nullclines for the state variables PU.1 and C/EBP in the case of no cytokine stimulation. The PU.1 nullcline is the locus of points where *d*[PU.1]/*dt* = 0, and the C/EBP nullcline is the locus of points where *d*[C/EBP]_T_/*dt* = 0. Where these nullclines intersect lie steady states of the full eight-dimensional set of ODEs. With no cytokine stimulation, these nullclines intersect five times to form three stable steady states (nodes) and two saddle points. The stable steady state with low levels of both C/EBP and PU.1 corresponds to a naïve GMP cell, whereas the other two stable steady states correspond to granulocyte and monocyte progenitor cells, depending on whether C/EBP or PU.1 is elevated, respectively. For the case of no cytokine stimulation, the GMP cell will sit indefinitely in the naïve state.

It is important to recognize that, in our model, “low” and “high” are relative. GMP cells are not typically described as having low concentrations of PU.1 and C/EBP, since both transcription factors are required for the transition of a common myeloid progenitor into a GMP cell (4, 33). In the framework of our model, however, it is appropriate to describe the GMP state as (PU.1_low_, C/EBP_low_), the granulocyte progenitor state as (PU.1_low_, C/EBP^high^), and the monocyte state as (PU.1^high^, C/EBP_low_). It is also important to note that, while PU.1 expression is elevated in neutrophils, PU.1 remains low in early granulocyte progenitors (71).

### M-CSF induces monopoiesis

We begin our investigation of external signaling by exploring how nullclines shift in response to M-CSF stimulation. Comparing Figures 4A,B, we see that, in response to M-CSF, the PU.1-nullcline moves and the naïve GMP state is lost. Although both the monocyte (PU.1^high^, C/EBP_low_) and granulocyte progenitor (PU.1_low_, C/EBP^high^) states remain, Figures 4B,C show that GMP cells under M-CSF stimulation preferentially differentiate into the monocyte state.

**Figure 5:**
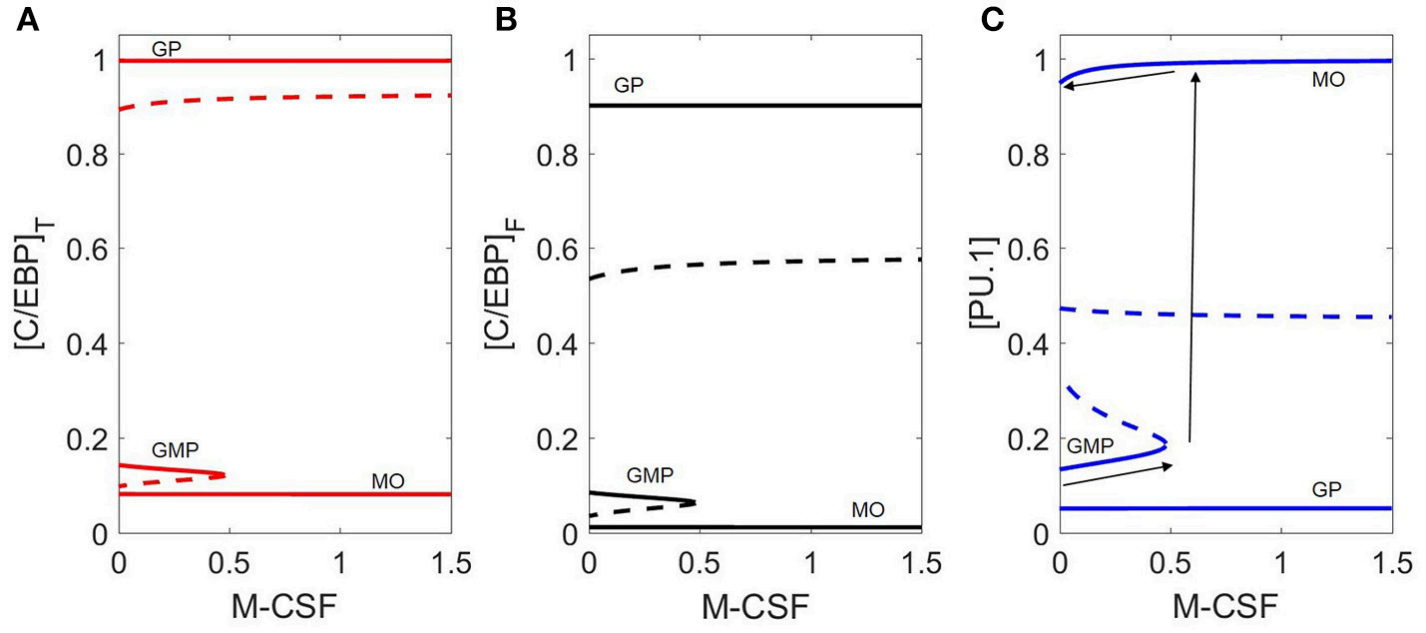
Bifurcation diagram with respect to M-CSF concentration. Three representations of the same bifurcation diagram, in terms of **(A)** [C/EBP]_T_, **(B)** [C/EBP]_F_, and **(C)** [PU.1]. Stable steady states are represented by solid lines and unstable states by dashed lines. The dynamical system undergoes a saddle-node bifurcation at [MCSF] ≈ 0.5, where the stable GMP state is lost. Black arrows on **(C)** demonstrates the irreversible switch of M-CSF induced monopoieses. The bottom line represents the change in the GMP state as [M-CSF] increases, the second arrow (pointing up) represents the “switch” in state and the third arrow (top) demonstrates that decreasing the M-CSF concentration does not return the cell to the GMP state.

Bifurcation diagrams with respect to M-CSF (Figure 5) illustrate how the cytokine affects the stability of GMP cells. We show (Figure 5C) how M-CSF concentration can destabilize the GMP state, resulting in differentiation into the monocyte state, and why this differentiation is irreversible. For cytokine concentrations less than ~0.5, there are three stable steady states, including one at [PU.1] ≈ 0.15, [C/EBP]_T_ ≈ 0.15 (the naïve GMP cell), whereas, for [MCSF] > 0.5, there are only two stable steady states, corresponding to monocytes and granulocyte progenitors. Thus, M-CSF induced monopoiesis is irreversible, as the monocyte state remains stable even if the M-CSF concentration is decreased to zero after the transition is made (see the black arrows in Figure 5C).

Although we use [C/EBP]_T_ and [PU.1] as primary markers of cell type, temporal changes in the other transcription factors (Figure 3A) give a more complete picture of the dynamics of the system. In the early stages of monopoiesis, we see an immediate increase in PU.1, IRF8, and Egr-2. IRF8 binds to C/EBP resulting in a slight decrease in C/EBP activity while Egr-2 represses Gfi-1, relieving suppression of PU.1. Furthermore, PU.1 upregulates itself, resulting in the switch-like behavior that is demonstrated in Figure 5C. Receptors such as GM-CSFR and M-CSFR are heavily upregulated while G-CSFR remains at a lower level (Figure 3C).

### G-CSF induces granulopoiesis

**Figure 6:**
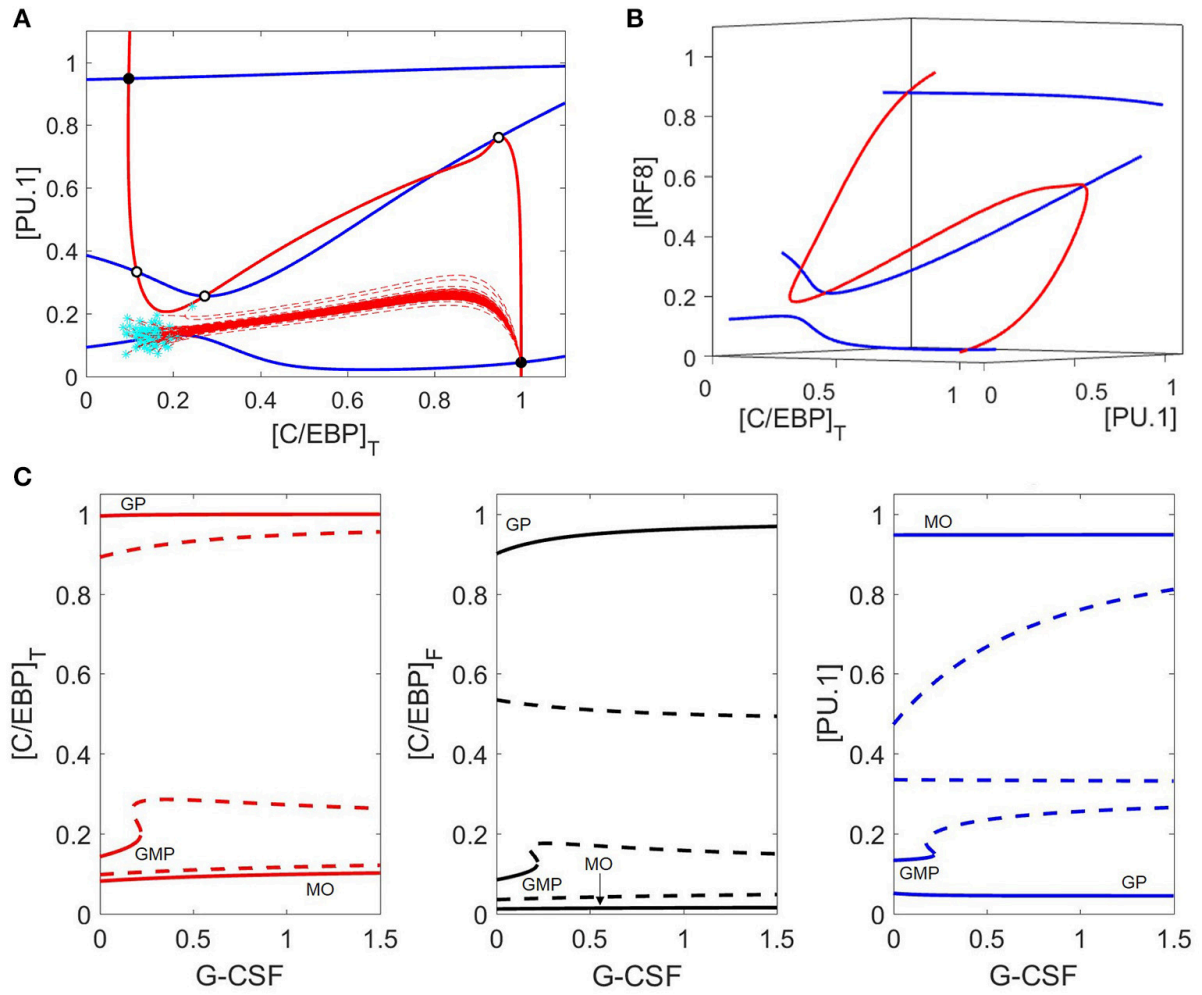
Phase plane and bifurcation in response to G-CSF. **(A)** Nullclines and trajectories as in Figure 4; [GCSF] = 1. Both PU.1 and C/EBP nullclines move in response to G-CSF, and only two stable steady states remain: the granulocyte state (bottom right) and the monocyte state (top left). GMP cells preferentially differentiate to the granulocyte state under G-CSF stimulation, as indicated by the dashed red lines**. (B)** Nullclines in a three-dimensional phase space {[PU.1], [C/EBP]_T_, and [IRF8]} reveal that there are only five steady states. The two other apparent nullcline intersections in panel **(A)** are artifacts of the projection onto a two-dimensional phase plane. **(C)** As in Figure 5, we show three different views of the same bifurcation diagram. Stable states are represented by solid lines and unstable states by dashed lines. For [GCSF] ≤ 0.2, the control system has three stable steady states (GMP, GP, MO) and two (or four) unstable steady states. For larger values of [GCSF], the GMP state is lost and only two stable steady states remain (GP and MO states).

G-CSF stimulation changes the landscape of the (PU.1, C/EBP) phase plane (Figure 6A) more drastically than M-CSF stimulation. Surprisingly, the PU.1 nullcline is more sensitive to changes in G-CSF than the C/EBP nullcline. As a result, there remain five intersection points, but only two are stable (the monocyte and granulocyte progenitor states). The other three steady states are two saddle points and an unstable node. There appear to be two additional intersection points of these nullclines; however, the apparent intersections are an artifact of projecting the nullclines onto the (PU.1, C/EBP) phase plane. By plotting the nullclines in a three-dimensional phase space in Figure 6B, we show that the nullclines intersect only five times.

The bifurcation diagram (Figure 6C) is in agreement with our nullclines, and shows that the GMP state disappears at [GCSF] ≈ 0.21, with only two stable steady states remaining (monocyte and granulocyte progenitor) and three unstable steady states. Despite the bistable nature of the system under G-CSF stimulation, GMP cells preferentially differentiate into granulocytes due to the locations of the basins of attraction of the two stable steady states (Figure 6A).

While experiments suggest that G-CSF induces granulopoiesis, the dynamical changes during this process of differentiation are not well documented. Our model (Figure 3B) suggests that G-CSF stimulation results in an initial increase of PU.1 expression, due to increased C/EBP activity, before PU.1 is eventually suppressed by Gfi-1. Egr-2 is also suppressed directly by Gfi-1, and IRF8 is suppressed when PU.1 activity decreases. The system reaches steady state as a granulocyte progenitor cell with high expression of C/EBP, Gfi-1 and G-CSFR, as well as moderate expression of GM-CSFR (Figure 3D).

Interestingly, when comparing the differentiation time of M-CSF induced monopoiesis and G-CSF induced granulopoiesis (Figures 3A,B), we find that GMP cells commit to the granulocyte progenitor state more quickly than to the monocyte state. This is likely a result of the fact that the auto-activation of C/EBP is stronger in our model than that of PU.1. It is known that it can take approximately 6 days for a monoblast (the earliest stage of monopoiesis) to mature into a monocyte, while it takes a GMP cell 1.5–2 days to mature into a promyelocyte (72, 73). As the transcription factor expression levels of our granulocyte progenitor state are similar with those of the promyelocyte state, we find that these temporal ratios are consistent with our simulations (71). However, we must note that, while these times are consistent with the literature, our model suggests that the differentiation time is concentration dependent (Figure S2).

### Low concentrations of GM-CSF favor monopoiesis while higher concentrations favor granulopoiesis

An important question we wish to address in this paper is: what possible mechanism can explain the concentration-dependent behavior of GM-CSF induced differentiation? GM-CSF signals upregulate C/EBP, which in turn promotes PU.1 and Gfi-1 transcription. However, Gfi-1 and PU.1 are mutually antagonistic, and PU.1 suppresses C/EBP activity via IRF8 (Figure 2B). Thus, C/EBP can inhibit PU.1 through Gfi-1, or suppress itself and Gfi-1 via activation of PU.1. We propose that this combination of positive and negative interactions that C/EBP has with PU.1, and the asymmetrical nature of the system, manifests itself in the concentration-dependent outcomes of GM-CSF induced GMP differentiation.

**Figure 7:**
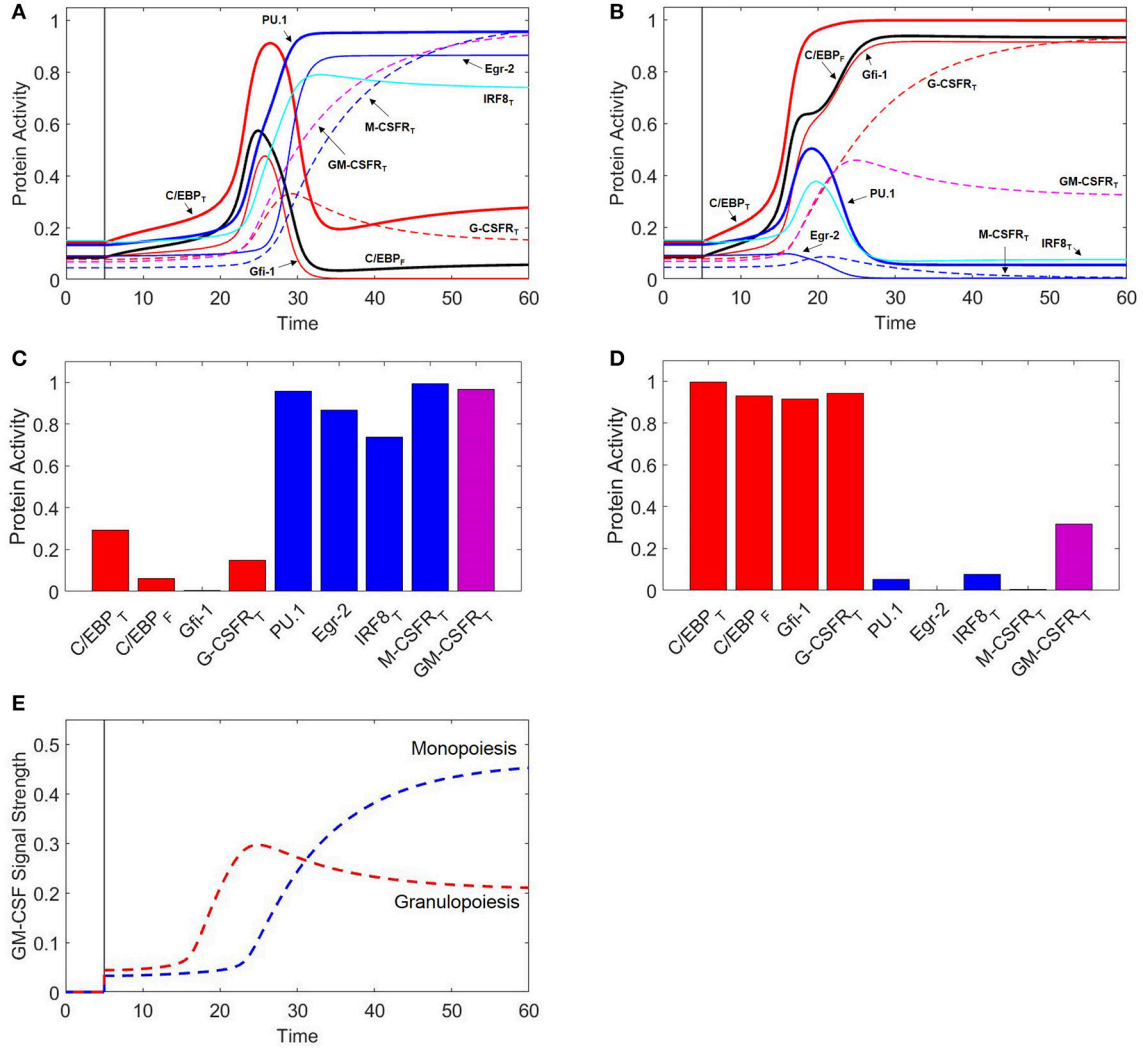
GM-CSF induces monopoiesis and granulopoiesis at low and high concentrations, respectively. When *t* ≤ 5, [GMCSF] = 0. When *t* > 5, [GMCSF] = 0.6 for low concentration **(A)** and 1.2 for high concentration **(B)**. Thick red line = [C/EBP]_T_, black line = [C/EBP]_F_, thick blue line = [PU.1], thin red line = [Gfi-1], thin blue line = [Egr-2], cyan line = [IRF8]_T_, dashed red line = [G-CSFR]_T_, dashed blue line = [M-CSFR]_T_, dashed magenta line = [GM-CSFR]_T_. **(C)** Final protein activity levels for simulation A indicate that low concentrations of GM-CSF induce monopoiesis. **(D)** Final protein activity levels for simulation B indicate that high concentrations of GM-CSF induce granulopoiesis. **(E)** GM-CSF signal strength during GM-CSF induced granulopoiesis and monopoiesis. Signal strength is proportional to and reported as [GMCSFR:GMCSF]. Blue dashed line represents a cell developing into a monocyte for [GMCSF] = 0.6. Red dashed line represents a cell developing into a granulocyte for [GMCSF] = 1.2. We find that higher concentrations of GM-CSF result in a higher initial signal to stimulate granulopoiesis; however, the signal decreases and levels off after the cell has committed to the granulocyte lineage. Lower concentrations of GM-CSF initially have lower signal strengths to initialize monopoiesis; however, GM-CSFR is upregulated to high levels after monocytic commitment, resulting in a greater GM-CSF signal strength in the monocytic lineage.

At low levels of GM-CSF (Figures 7A,C), both C/EBP and PU.1 rise swiftly. PU.1's positive autoregulation drives it to increase faster than Gfi-1, promoting IRF8 and Egr-2 production in the process. IRF8 binds to and suppresses C/EBP, preventing C/EBP-induced expression of Gfi-1, while Egr-2 directly suppresses Gfi-1. Eventually, Gfi-1 is irreversibly suppressed and PU.1 is dominant. The resulting phenotype resembles that of the monocyte lineage. Thus our model agrees with experimental observations, that low concentrations of GM-CSF encourage monopoiesis (9). When we compare M-CSF and GM-CSF induced monopoiesis (Figures 3A, 7A), we find that the final expression patterns are very similar, however the evolution of transcription factor expression is different. Notably, during GM-CSF induced monopoiesis, C/EBP levels and Gfi-1 levels rise substantially prior to being suppressed, while C/EBP and Gfi-1 remain low in M-CSF induced monopoiesis. Our model also suggests that GM-CSF induced monopoiesis is more rapid than M-CSF induced monopoiesis (Figure S2).

At higher concentrations of GM-CSF (Figures 7B,D), C/EBP increases more rapidly due to a combination of stronger GM-CSF stimulation, suppression of IRF8, and C/EBP positive autoregulation. The rapid increase in C/EBP results in acceleration of Gfi-1 production. While PU.1 expression is also enhanced, PU.1 relies heavily on its own capacity to auto-activate. Therefore, when C/EBP is increased, there is a delay before PU.1 reaches its maximum production rate; however, Gfi-1 reaches its maximum production rate immediately. Thus, Gfi-1 is more responsive to a change in C/EBP than PU.1. Furthermore, Gfi-1 directly suppresses PU.1 and Egr-2, while PU.1 must upregulate Egr-2 to inhibit Gfi-1. If Gfi-1 increases faster than PU.1, it halts PU.1-induced Egr-2 expression and establishes dominance over PU.1. In this way, our model predicts that high concentrations of GM-CSF will induce granulopoiesis over monopoiesis, a result which is consistent with experimental observations (9).

We find that, even though the differentiation times of GM-CSF and G-CSF induced granulopoiesis are very similar (Figure S2), during GM-CSF induced granulopoiesis the PU.1 and IRF8 levels spike considerably higher than during G-CSF induced granulopoiesis (Figures 3B, 7B). This is likely due to greater Gfi-1 activity during earlier stages of G-CSF induced granulopoiesis.

Intriguingly, our model suggests that GM-CSFR expression decreases slightly after granulocytic commitment, and remains at lower levels than the monocytic lineage. Experimental evidence shows that, indeed, GM-CSFR expression is higher in monocytes than in granulocytes, despite the fact that higher concentrations of GM-CSF favor granulopoiesis over monopoiesis (9, 74, 75). To explore why, we examine the incoming signal strength of GM-CSF over time with high and low GM-CSF concentrations (Figure 7E). We find that the incoming GM-CSF signal is stronger in the short term under high-dose conditions (granulopoiesis), however the signal strength begins to decrease after ~24 time units due to reduced GM-CSFR expression. In contrast, at lower doses of GM-CSF (monopoiesis), the signal strength remains low until ~24 time units, when it increases substantially in a hyperbolic fashion to levels much higher than in granulopoiesis. We propose that the sudden increase in signal is due to a switch-like mechanism, resulting from the positive feedback loop involving GM-CSFR, C/EBP and PU.1. As a result of this mechanism, we observe that the lower the GM-CSF concentration, the longer it takes for the switch to kick in. We ascertain that, at low GM-CSF concentrations, the delay in the switch event permits PU.1 to establish dominance over Gfi-1 and C/EBP, and commit to the monocyte lineage. The signal strength of GM-CSF is half-maximal at ~30 time units after stimulation. At this point in monopoiesis (Figure 7A), Gfi-1 is subdued, C/EBP is on a steep decline, and monocytic transcription factors are highly expressed. Thus, by the time the GM-CSF signal is strong, the cell is already committed to the monocyte lineage. Similarly, with higher levels of GM-CSF, the cell has decisively committed to the granulocytic lineage at the point of maximum signal strength (~24 time units in Figure 7B).

Our results suggest that in both monopoiesis and granulopoiesis the GM-CSFR signaling capacity changes significantly after the cell has already committed to one lineage over another. If this is true, then the high concentration of GM-CSFR in monocytes relative to granulocytes must serve an alternative function than lineage commitment. One possibility is that GM-CSFR signaling, or lack thereof, is crucial for regulating proteins not accounted for by this model. Alternatively, GM-CSF signaling may function to upregulate C/EBP in the monocytic lineage, since it is necessary for AP-1 to bind with C/EBP to promote monocytic genes (76). It is also possible that downregulation of GM-CSFR is crucial for proper granulocyte development, as C/EBPα is downregulated in later stages of granulopoiesis (77). While future experimental studies may clarify these issues, our model does lead us to an additional conclusion that we will discuss in the subsequent section.

### GM-CSF induces M-MDSC differentiation

**Figure 8:**
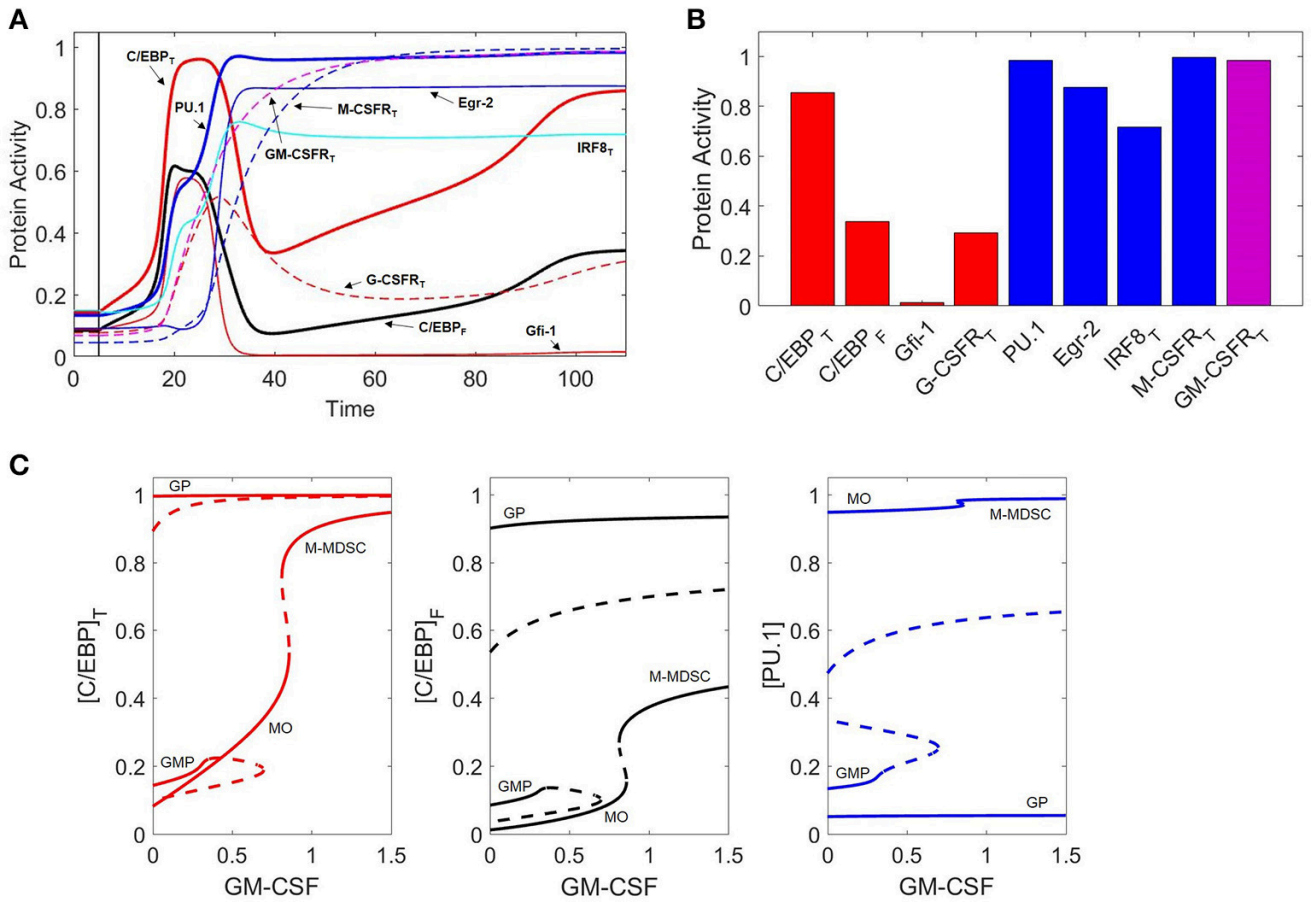
GM-CSF induces M-MDSC differentiation. **(A)** Protein activity over time (*t*) during GM-CSF induction of the M-MDSC phenotype. When *t* < 5, [GMCSF] = 0 units. When *t* > 5, [GMCSF] = 0.9. Line descriptions are the same as Figures 3, **7A,B**. **(B)** Final protein activity levels for simulation A indicate that moderate concentrations of GM-CSF can induce the M-MDSC phenotype. **(C)** Three views of the same bifurcation diagram. Stable states are represented by solid lines and unstable states by dashed lines. At [GMCSF] = 0, the system exhibits three stable nodes (GMP, granulocyte progenitor, and monocyte) and two unstable saddle points. At [GMCSF] ≈ 0.35, the GMP state is destabilized, and it briefly re-stabilizes for 0.66 < [GMCSF] < 0.69. The MO state (PU.1^high^, C/EBP_low_) is stable until [GMCSF] ≈ 0.86, at which point it switches to the M-MDSC state (PU.1^high^, C/EBP^high^).

If low levels of GM-CSF induce monopoiesis and high levels induce granulopoiesis, what happens when we try something in the middle? Remarkably, our model predicts that moderate exposure to GM-CSF can induce GMP differentiation into a hybrid state: PU.1^high^ C/EBP^high^ (Figures 8A,B). Moreover, we find that the dynamics of this process are strikingly similar to GM-CSF-induced monopoiesis. While C/EBP and PU.1 both rise swiftly early in the process, there is a lag in GM-CSFR expression, allowing PU.1 to establish dominance over C/EBP and Gfi-1. Thus, the cell begins to resemble the monocytic phenotype. However, when GM-CSFR approaches maximum expression, the signal becomes strong enough to induce a switch in C/EBP behavior, resulting in high C/EBP expression. Furthermore, a large fraction of C/EBP binds with IRF8, restricting its capacity to activate granulocytic genes. As a result of this and high levels of Egr-2, Gfi-1 remains repressed. The outcome is a new hybrid state (PU.1^high^, C/EBP^high^). Naturally, the question arises, is there a myeloid cell that fits this description? Indeed, M-MDSCs fit this profile, as these monocytic cells presumably express high levels of PU.1 and are known to highly express C/EBPβ (78). Furthermore, M-MDSCs highly express IRF8 relative to granulocytes, and are likely to express high levels of Egr-2 and low levels of Gfi-1 as these are mutually antagonistic master regulators of the monocytic and granulocytic lineages, respectively (15, 61). Because the hybrid state fits the expected expression profile of M-MDSCs and displays behavioral characteristic observed in M-MDSC experiments (as discussed below), we propose that this hybrid state is representative of M-MDSCs and refer to this state as the M-MDSC state for the remainder of the paper.

We have described three distinct expression profiles that result from different GM-CSF concentrations, but it is still unclear which phenotypes are favored over the entire range of GM-CSF concentrations. To evaluate this “favorability spectrum,” we simulated 10,000 stochastically generated cells under different GM-CSF conditions (Table 1). The results confirm that the monocytic state is heavily favored at lower concentrations of GM-CSF. However, the population ratio shifts toward granulocytes as the dose of GM-CSF is increased. We also observe a distinct dichotomy in the expression of monocytes and M-MDSCs, suggesting that GM-CSF induces some kind of toggle switch.

**Table 1:** Population ratios over a range of GM-CSF concentrations.

**[GMCSF]**	**% Naïve GMP**	**% Granulocyte Prog**.	**% Monocyte**	**% M-MDSC**
0	99.79	0.2	0.01	0
0.2	99.21	0.48	0.31	0
0.4	0	4.15	95.85	0
0.6	0	18.21	81.79	0
0.8	0	38.57	61.43	0
1.0	0	53.97	0	46.03
1.2	0	62.88	0	37.12
1.4	0	68.20	0	31.80

*We simulated the differentiation of 10,000 stochastically generated cells at increasing GM-CSF concentrations over a time period of 150 time units (~300 h). We classified the final state as “naïve GMP,” “granulocyte progenitor,” “monocyte” or “M-MDSC.” The results show that monocytes are heavily favored at low concentrations of GM-CSF, while granulocytes are favored at high concentrations. Monocyte differentiation yields to the M-MDSC phenotype at higher GM-CSF concentrations*.

To explore these effects further, we computed one-parameter bifurcation diagrams with respect to GM-CSF concentration (Figure 8C). Indeed, we find that a toggle switch (saddle-node bifurcation) does occur from the monocyte state to the hybrid state when [GMCSF] ≈ 0.86. This suggests that the monocyte state is unstable at high GM-CSF concentrations, while the M-MDSC state is dependent on significant cytokine stimulation.

**Figure 9:**
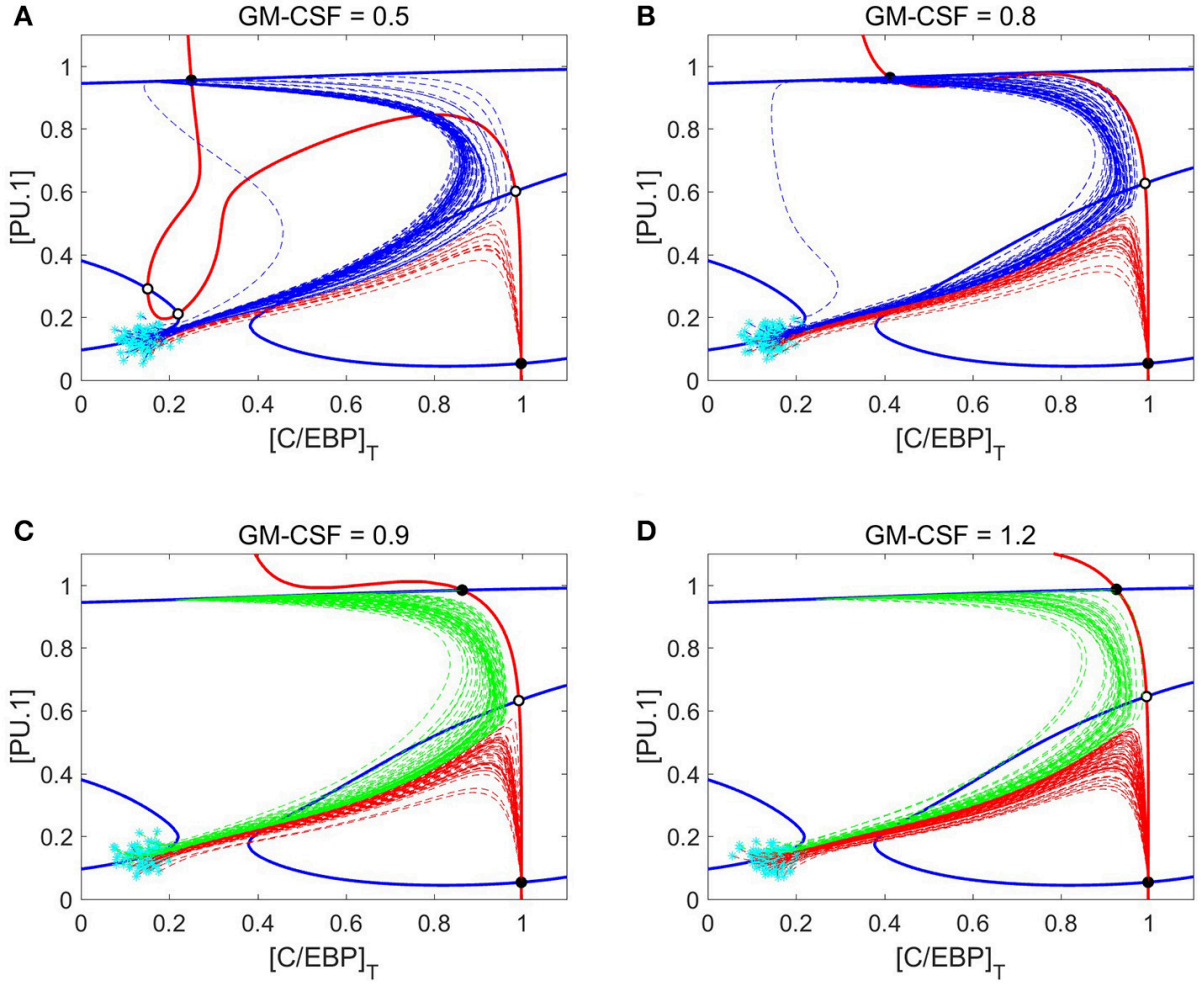
Nullcline shifts, in response to changes in GM-CSF concentration, illustrate the switch from monocyte state to M-MDSC. **(A–D)** Phase planes at four different concentrations of GM-CSF: 0.5, 0.8, 0.9, and 1.2. Solid blue and red lines denote the PU.1 and C/EBP nullclines, respectively. Black and white circles designate stable and unstable steady states, respectively. Cyan asterisks represent stochastically generated initial conditions for a population of simulated cells. After stimulation by GM-CSF, the cells follow the dotted lines (trajectories of the full eight-dimensional system of ODEs projected onto the two-dimensional phase plane). Blue dotted lines end up at a monocyte steady state, red lines at a granulocyte progenitor steady state, and green lines at an M-MDSC steady state. As [GMCSF] increases, the C/EBP nullcline moves in such a way that the monocyte state gives way to a new M-MDSC state.

To better understand the dynamics of cell differentiation at varying GM-CSF concentrations, it is helpful to consider the phase planes and cell trajectories in Figure 9. We find that the PU.1 nullcline does not respond to GM-CSF; however, the C/EBP nullcline moves in such a way that the granulocyte state remains fixed in position and the monocyte state shifts substantially. In agreement with the bifurcation diagrams, the nullclines show that, as [GMCSF] increases, the monocyte state moves toward higher concentrations of C/EBP. Furthermore, with this changing nullcline landscape, the basins of attraction alter, resulting in a shift of favorability toward the granulocyte progenitor state. The representative cell trajectories (dashed lines in Figure 9) are good indicators of how the nullcline shifts affect cell differentiation. At [GMCSF] ≈ 0.86, the C/EBP nullcline lifts away from the PU.1 nullcline, so that the monocyte state disappears and the M-MDSC state is revealed.

Figure 9 shows that M-MDSC differentiation follows a similar trajectory as GM-CSF induced monopoiesis, as we observed before when comparing Figures 7A, 8A. The pattern of monocyte differentiation is particularly interesting. The differentiating cells move in an arching fashion, first toward states of high PU.1 and C/EBP and then toward states of high PU.1 and low C/EBP; overshooting the monocyte steady state, they make a second turn-around, involving increasing concentration of C/EBP, as they approach the stable monocyte steady state. This pattern is seen in Figures 7A, 8A as well, where the C/EBP concentration rises (the arching phase), plummets (the passing phase) and then begins to rise again (the second-turn phase). Differentiation dynamics of the M-MDSC phenotype are quite similar, the critical difference being that the final steady state has much larger concentrations of C/EBP than is typical of monocyte cells.

These results suggest that the stability of the monocyte and M-MDSC states is dependent on the extracellular GM-CSF concentration. Thus, a monocyte exposed subsequently to higher levels of GM-CSF should transition into the M-MDSC state. This result is consistent with experiments that suggest tumor-conditioned media can convert monocytes into M-MDSCs and that GM-CSF can induce M-MDSC differentiation from myeloid progenitors (31, 79, 80). Similarly, the model suggests that M-MDSCs that are removed from GM-CSF stimulus should be destabilized. Figure S3 explicitly shows how these transitions can occur. We find that the ability of GM-CSF to convert monocytes into M-MDSCs is partially due to high expression of GM-CSFR within the monocyte lineage, as the signal strength must be sufficiently high to induce this transformation. This suggests one possible biological motivation for monocytes to express such high levels of the receptor, as this monocytic plasticity may be useful in a variety of pathological conditions.

### Combined treatment with G-CSF and M-CSF results in a heterogeneous population of granulocytes and monocytes

**Figure 10:**
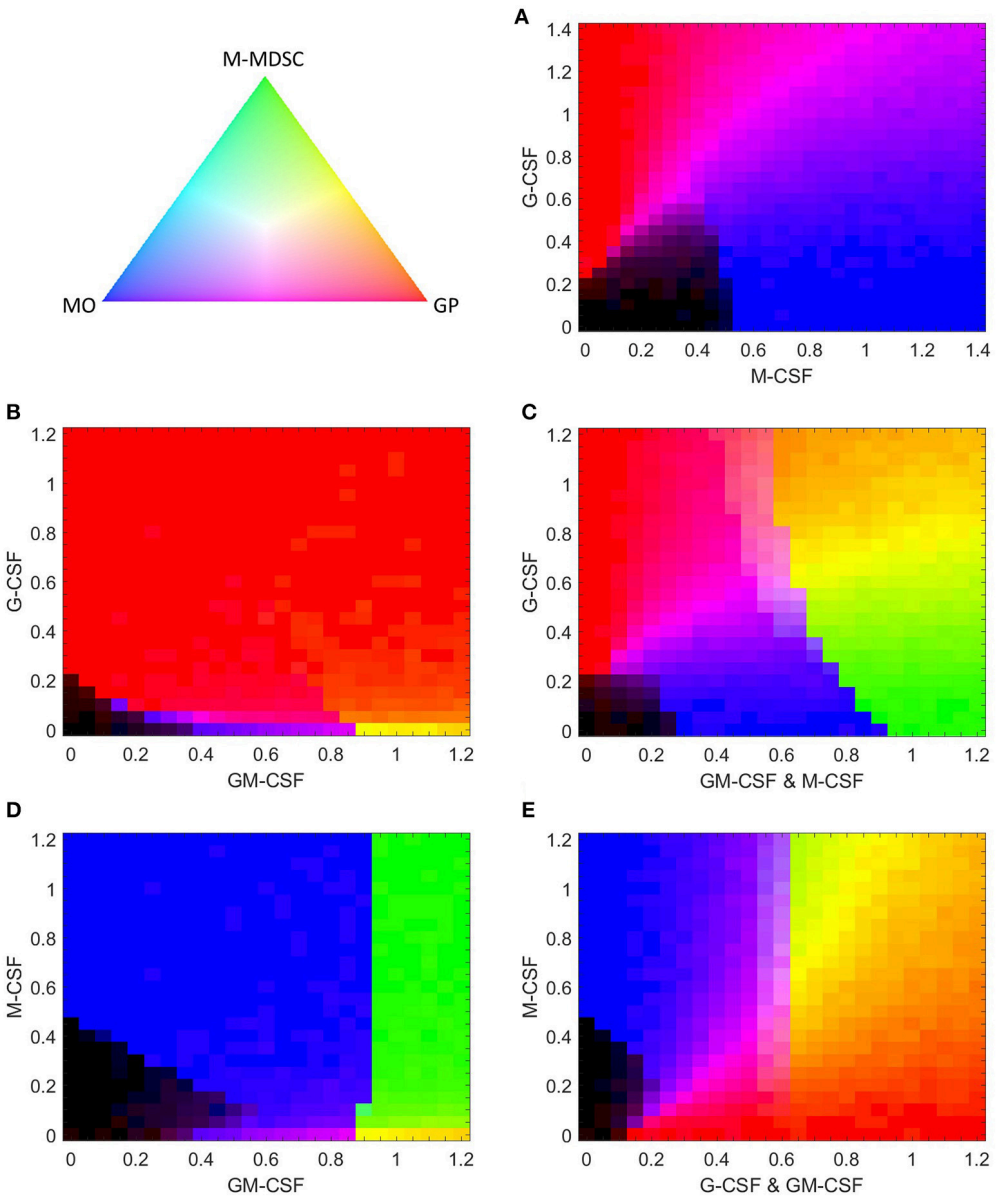
Simulated composition of stochastically generated populations of cells in response to various combinations of cytokines. Each cytokine combination is simulated with 500 stochastically generated cells. The color gradient triangle on the top left correlates to population composition. Populations containing all M-MDSCs, granulocyte progenitors (GP), or monocytes (MO) are represented by green, red and blue, respectively. Black represents populations of undifferentiated cells. The mathematical relationship between population ratios and pixel color is discussed in “computational methods”. **(A,B)** G-CSF paired with M-CSF or GM-CSF. **(C)** G-CSF paired with equal concentrations of GM-CSF and M-CSF. **(D,E)** M-CSF paired with GM-CSF or with equal concentrations of G-CSF and GM-CSF.

If G-CSF and M-CSF promote granulopoiesis and monopoiesis, respectively, what happens when we expose a cell to both simultaneously? A heat map of M-CSF and G-CSF stimulation (Figure 10A) suggests that M-CSF may inhibit granulopoiesis at lower concentrations of G-CSF. However, when both cytokines are introduced at higher levels, our model suggests that the resulting population will be a heterogeneous mix of both granulocytes and monocytes, a result in agreement with experimental observations (8). Surprisingly, the model suggests that GMP cells stimulated by both G-CSF and M-CSF never differentiate into M-MDSCs. Phase plane analysis suggests that the M-MDSC state does not exist under such conditions (Figure S4).

### G-CSF can push cells toward monopoiesis in low signaling conditions

An intriguing phenomena occurs when G-CSF is paired with low doses of M-CSF. Figure S5 (an alternative view of the lower-left corner of Figure 10A) shows that G-CSF can induce monopoiesis at M-CSF concentrations too weak to stimulate differentiation alone. Although several cells differentiate into granulocytes under these conditions, the fact remains that a larger percent of GMP cells differentiate into monocytes than if G-CSF were absent. G-CSF has a similar effect when paired with GM-CSF. Figure S6 (an expanded view of the lower-left corner of Figure 10B) shows that low concentrations of G-CSF can actually lower the GM-CSF dose required to induce monopoiesis. When [GCSF] = 0, significant monocytic development is not triggered until [GMCSF] > 0.35; however, with [GCSF] = 0.05, the required minimal dose of GM-CSF decreases to 0.2. When the concentration of G-CSF is increased further, however, it pushes the system toward granulopoiesis. Ultimately, these results suggest that, for cells that are primed for the monocyte lineage but do not have quite enough stimulus to initiate the process, G-CSF can provide the small push that is necessary to initiate monopoiesis. However, if G-CSF is introduced at higher concentrations, it will induce granulopoiesis at the expense of monopoiesis.

### G-CSF can inhibit or encourage M-MDSC development when paired with M-CSF and GM-CSF

Having evaluated the effects of G-CSF coupled with M-CSF and GM-CSF individually, we naturally progress to evaluate G-CSF effects when paired with equal signals from GM-CSF and M-CSF (GM/M-CSF). As one might expect, our model predicts that, when paired with low levels of GM/M-CSF, G-CSF can induce GMP cells to favor granulopoiesis over monopoiesis (Figure 10C). However, at higher levels of GM/M-CSF that still favor the monocyte phenotype {the interval [0.65, 0.9] in Figure 10C}, G-CSF can push the system in favor of M-MDSC development. In fact, the closer the GM/M-CSF signal is to the M-MDSC switch threshold (≈0.9), the less G-CSF is required to induce the M-MDSC phenotype. The capacity for G-CSF to induce GMPs to differentiate into M-MDSCs suggests that already differentiated monocytes in similar conditions (with GM-CSF) can also be pushed into the M-MDSC state by G-CSF. These results are intriguing, as G-CSF is typically associated with its effects on granulopoiesis and PMN-MDSCs, rather than M-MDSCs (31, 81). Furthermore, we find that, in conditions that favor M-MDSCs, additional G-CSF can push GMP cells in favor of granulopoiesis. Therefore, our model predicts that G-CSF can either promote or inhibit the production of M-MDSCs, depending on the extracellular conditions.

### M-CSF can induce M-MDSC differentiation when mixed with GM-CSF

It has been well documented that both M-CSF and GM-CSF can contribute to M-MDSC development (82, 83). Since our model indicates that M-CSF alone cannot induce the M-MDSC phenotype, we test to see what effect it has when coupled with GM-CSF (Figure 10D). We find that a GM-CSF primed system is hyper-sensitive to M-CSF, as even very low doses of M-CSF can arrest granulopoiesis to favor M-MDSCs. (For detailed effects on granulopoiesis, see Figure S7.) This suggests that the effects of M-CSF are minimally concentration-dependent. We suspect that this extreme sensitivity is unrealistic for real-life conditions, as the sensitive behavior would likely be washed out by other disturbances, such as other cytokines *in vivo* or in growth serums. Regardless, this calculation suggests that M-CSF paired with GM-CSF makes for a much stronger inducer of the M-MDSC phenotype than either cytokine alone.

Finally, we evaluate GMP behavior when M-CSF is paired with equal concentrations of G-CSF and GM-CSF (GM/G-CSF) (Figure 10E). We find that, under conditions that would otherwise encourage granulopoiesis, M-CSF can induce both monocytes and M-MDSCs. In contrast, when M-CSF was exclusively paired with G-CSF, M-MDSCs are not produced (Figure 10A). Furthermore, Figure 10E is at odds with Figure 10D, where the effects of M-CSF with GM-CSF alone are not concentration dependent. However, as M-CSF is increased in a GM/G-CSF system, the proportion of M-MDSCs increases in a concentration-dependent manner. We suggest that the situation in Figure 10E is more realistic for experimental and biological settings than Figure 10D, as the concentration-dependent behavior is likely more robust to biological disturbances.

## Discussion

In consideration of the crucial roles played by cells of the GMP lineage in human health and disease, we have proposed a molecular regulatory network for the differentiation of GMP cells (Figure 2B), based on known facts about the underlying molecular controls of this aspect of hematopoiesis. From our proposed network we have constructed a dynamical model of GMP cell lineage commitment (see Supplementary Material for a complete specification of the mathematical model), and we have used numerical simulations and bifurcation analysis to reveal the dynamical consequences of the model. The simulated responses of the model to cytokine stimuli are in good agreement with the fundamental characteristics of GMP differentiation: that G-CSF and high concentrations of GM-CSF favor granulopoiesis, while M-CSF and lower concentrations of GM-CSF favor monopoiesis (8–10). Furthermore, the model makes several intriguing predictions concerning progenitor cell responses to CSF signals. For an itemized list of model predictions, see the Supplementary Material.

### Concentration-dependent effects of GM-CSF signaling on GMP differentiation

Investigating the concentration-dependent response of GMP cells to GM-CSF, we uncovered three main features of the response. First: the dual regulatory effects of C/EBP on PU.1; C/EBP induces PU.1 directly by promoter stimulation and inhibits PU.1 indirectly through stimulation of Gfi-1 (40–43). The balance of these effects is dependent on the concentration of GM-CSF. At high concentrations of GM-CSF, C/EBP increases quickly, resulting in a swift rise in Gfi-1 and repression of PU.1, thereby inducing granulopoiesis. The result, that C/EBP is an antagonist of PU.1 in granulopoiesis, is in agreement with Wang et al. (84), who showed that induction of an isoform of C/EBPα downregulates the *SPI1* gene (encoding PU.1) to promote granulopoiesis. Alternatively, our model predicts that C/EBP has a positive impact on PU.1 in GM-CSF induced monopoiesis, as a result of a slower increase in C/EBP, allowing PU.1 enough time to upregulate itself and establish dominance. Second: PU.1's indirect antagonism of C/EBP and Gfi-1 is essential to commit the cell toward monopoiesis. Third: GM-CSFR signaling forms positive feedback loops with PU.1 and C/EBP. When stimulated, GM-CSFR transmits a signal to C/EBP to increase both C/EBP and PU.1 expression. These proteins, in turn, upregulate GM-CSFR resulting in a stronger GM-CSF signal, which results in even greater stimulation of C/EBP.

These feedback loops create a sensitive, switch-like response of gene expression to GM-CSFR stimulation. We find that the lower the GM-CSF concentration is the longer it takes for the switch to kick in. In GM-CSF-induced granulopoiesis, the switch kicks in early, to allow sufficient upregulation of C/EBP and Gfi-1. In GM-CSF-induced monopoiesis, the switch is delayed, to allow PU.1 to upregulate itself and repress C/EBP and Gfi-1. In this way, we propose that these three dynamic features of the control system work synergistically to produce the unique behaviors associated with GM-CSF-induced differentiation.

### Differences among CSF-induced differentiation processes

Given that our model successfully captures the endpoints of GMP differentiation induced by G-, M-, and GM-CSF, we propose that our model can also offer significant insights into the different temporal patterns of protein concentrations during the differentiation processes. For example, GM-CSF induced monopoiesis exhibits a significant spike in C/EBP and Gfi-1 concentrations in its early stages, followed by suppression of both proteins, whereas M-CSF induced monopoiesis does not exhibit such a spike. It is possible that these differences could influence downstream transcription factors not accounted for in our model, perhaps resulting in different subtypes of monocytes. (Alternatively, these incongruences may be short-lived, making no difference on the final phenotype.) Nonetheless, our model predicts that the final concentration of C/EBP in monocytes is dependent on the signaling strength of GM-CSF (see the MO branch in Figure 8C). Since the subtype of the monocyte may well depend upon its level of expression of C/EBP, the concentration of GM-CSF in the micro-environment of a differentiated monocyte may have immediate implications on the phenotype of the cell. Intriguingly, it has been shown that GM-CSF induced monocyte-derived macrophages are distinctly different in genetic expression from M-CSF induced monocyte-derived macrophages (85, 86). Perhaps GM-CSF's influence on C/EBP concentration in this lineage plays some role in the differences observed in these macrophages. In addition, our model suggests that monopoiesis may be induced more quickly by GM-CSF than by M-CSF. If true, GM-CSF may be better suited for emergency monopoiesis than M-CSF.

Similarly, we find that GM-CSF induced granulopoiesis exhibits a larger spike in PU.1 and IRF8 concentrations in its early stages than G-CSF induced granulopoiesis. Although these differences are not as dramatic as the differences between M-CSF and GM-CSF induced monopoiesis, we cannot dismiss the possibility that these differences may effect downstream transcription factors and prime the cells for different subtypes of granulocytes. For example, it has been shown that GM-CSF has a higher propensity for inducing eosinophils than G-CSF (8, 87).

## GM-CSFR expression patterns of myeloid cells

An unexpected finding of our model, which agrees with experimental data, is that cells of the granulocyte lineage express lower concentrations of GM-CSFR than monocytes (74, 75). This is counter-intuitive, as granulocytic differentiation is favored over monocytes at higher concentrations of GM-CSF (9). Our model suggests that the signal strength of GM-CSFR is stronger in the initial commitment step of GM-CSF-induced granulopoiesis when compared to monopoiesis. However, after the lineage fate is fixed, the concentration of GM-CSFR continues to increase in monopoiesis, but decreases slightly in granulopoiesis (Figure 7E). We suspect that these conditions may be crucial for cellular maturation. It is possible that lower levels of GM-CSFR are required to prevent excessive stimulation of C/EBPα, as C/EBPα is downregulated in later stages of granulopoiesis (77). It is also possible that high GM-CSFR expression is important in later stages of monopoiesis, to stimulate C/EBP. It is known that C/EBP not only stimulates PU.1, but forms a complex with AP-1 in monocytes to activate monocytic genes rather than granulocyte genes (40, 76). Thus, the capacity to receive a strong GM-CSF signal may be important for gene regulation within the monocytic lineage. In agreement with this hypothesis, our results suggest that expression of C/EBP in monocytes increases as the GM-CSF concentration increases.

### Dynamics of the monocytic myeloid-derived suppressor cell

Our model predicts that the differentiation dynamics of M-MDSCs is very similar to typical monopoiesis; however, once the cell has committed to the monocytic lineage, there is a substantial upregulation of C/EBP. Since M-MDSCs have been shown to express high concentrations of C/EBPβ, but not C/EBPα, we suspect that some mechanism not captured by our model selectively suppresses C/EBPα (78). We get by without this mechanism, as the function of C/EBPβ and C/EBPα are redundant in hematopoiesis (35). Our model suggests that a significant fraction of C/EBP in M-MDCSs (and monocytes) is bound to IRF8, suggesting that its impact on granulocytic genes is diminished in these cells. Just like monocytes, the model suggests that PU.1, IRF8, Egr-2, M-CSFR, and GM-CSFR are all expressed at high levels in M-MDSCs as well, while G-CSFR is expressed at levels somewhere between a monocyte and a granulocyte progenitor. Thus, the G-CSFR is potentially a usable marker to distinguish between monocytes and M-MDSCs. Of course, if the variance of G-CSFR expression is large in monocytes or M-MDSCs, G-CSFR will not be an effective marker. Regardless, this suggests that G-CSF may have a stronger influence on M-MDSCs than on monocytes.

Intriguingly, our model suggests that high GM-CSF concentrations can induce a monocyte to morph into an M-MDSC. This behavior is a consequence of high expression of GM-CSFR in monocytes. Additionally, our results suggest that the stability of this M-MDSC state is dependent on GM-CSF stimulation. Thus, if an M-MDSC is removed from cytokine stimulation, the phenotype of the cell will change. These results agree with the literature, as monocytes can be programmed into M-MDSCs in tumor microenvironments, and terminally differentiate into macrophages and dendritic cells when removed from stimulatory conditions (31, 32). However, as our model is not designed to simulate terminal differentiation into macrophages and dendritic cells, it predicts that M-MDSCs will revert back into monocytes when GM-CSF is withdrawn. We hypothesize that M-MDSCs can be destabilized via the mechanism of our model, but rather than reverting back to a monocyte, will terminally differentiate due to other variables not accounted for in our model.

### CSF synergies and crosstalks

We find that G-CSF may play a more dynamic role in GMP differentiation than has been previously proposed. G-CSF is typically thought of as an inducer of the granulocyte lineage, but our model suggests that GMP cells likely exhibit an entire spectrum of differentiation behaviors in response to G-CSF due to signaling crosstalk. We find that, at concentrations of M-CSF not quite sufficient to induce monopoiesis, small concentrations of G-CSF can provide the nudge necessary to initiate monocytic differentiation. We see a similar phenomenon when G-CSF is introduced with GM-CSF: if a cell is primed for monopoiesis, a small concentration of G-CSF may provide the stimulus needed to induce monopoiesis. However, when G-CSF is increased to higher concentrations, monopoiesis is arrested in favor of granulopoiesis. The model also predicts that G-CSF can induce M-MDSC development under the right conditions. Our simulations suggest that normal monopoiesis, in response to simultaneous stimulation by a combination of moderate levels of M- and GM-CSFs, can be skewed in favor of M-MDSCs if paired with G-CSF (see Figure 10C). This also suggests that G-CSF can induce monocytes in such conditions to differentiate into M-MDSCs. On the other hand, under conditions that normally favor M-MDSC development, higher G-CSF concentrations will push differentiation in favor of granulopoiesis. Therefore, our model suggests that G-CSF can either promote or inhibit M-MDSC differentiation, depending on extracellular conditions. These predictions should be tested in a laboratory environment, as the implications are far reaching. It is possible that low levels of G-CSF may be utilized *in vivo* to aid monopoiesis and M-MDSC development.

Contrary to the dynamic role of G-CSF, our model suggests that M-CSF plays an exclusively antagonistic role in granulopoiesis. We predict that, under conditions of low G-CSF concentration, M-CSF can interfere with granulopoiesis to arrest GMP differentiation. We also find that M-CSF may drive M-MDSC differentiation under conditions that would normally favor granulopoiesis, depending on the relative concentrations of GM-CSF and G-CSF. Furthermore, our model suggests that pairing high concentrations of M-CSF and GM-CSF may be a potent inducer of M-MDSCs.

### CSFS as clinical targets

Cumulatively, our results suggest that M-CSF, GM-CSF, and G-CSF can all favor M-MDSC development, depending on extracellular conditions. We suspect that high concentrations of M-CSF and GM-CSF, as well as lower concentrations of G-CSF, may be present in some biological environments that support M-MDSC development, such as a tumor micro-environment. Indeed, several tumors associated with MDSCs have been reported to express M-CSF, GM-CSF, and/or G-CSF (18). We propose that a model such as ours can be used to explore the effects of tumor-specific conditions on hematopoiesis. For instance, our model suggests that G-CSF may contribute to M-MDSC differentiation under some, but not all, conditions that are otherwise favorable to monocyte differentiation. Thus, inhibiting G-CSF may be a successful strategy to destabilize the M-MDSC state in a tumor micro-environment where G-CSF is expressed alongside M-CSF and GM-CSF. However, while G-CSF's role in M-MDSC development is more context dependent, our results suggest that M-CSF and especially GM-CSF signaling act as much stronger inducers of M-MDSCs. Interestingly, while GM-CSF may induce M-MDSCs independent of other CSFs, the model suggests that G-CSF and M-CSF are reliant on GM-CSF to induce the M-MDSC state. Therefore, we propose that, among the CSFs, GM-CSF is the most promising therapy target for M-MDSC-associated tumors, while M-CSF may be an excellent alternative. In agreement with our results, knockdown of tumor-released GM-CSF in mice significantly reduced M-MDSC populations, and resulted in increased anti-tumor suppressive immunity (79). In another study, inhibiting M-CSFR signaling suppressed M-MDSC populations, while making no difference to the PMN-MDSC population. Furthermore, when paired with the VEGFR-2 antibody, blocking M-CSFR signaling resulted in a significant reduction in tumor angiogenesis (25). In both instances, the ratio of PMN-MDSCs to M-MDSCs increased, suggesting that these effects are due, in part, to altered differentiation rather than proliferation.

Alternatively, since MDSCs may be useful in a variety of pathological conditions, such as sepsis and burns (22, 88), an effective therapeutic strategy may be to upregulate M-MDSCs by administering a combination of GM-CSF and M-CSF (see Figures 10C,**D**). We acknowledge that *in vivo* other cytokines that are similar to GM-CSF (such as IL-3) may play comparable roles in M-MDSC differentiation (89). Thus, M-CSF and G-CSF may still influence M-MDSC differentiation under conditions where GM-CSF is absent, increasing their value as therapeutic targets.

### Network topology

Ultimately, the behavior of the model is a consequence of the network topology, i.e., multiple feedback and feedforward loops in the reaction mechanism (Figure 2B) and the relative strengths of these interactions (e.g., the ω_*i, j*_'s in our mathematical model). For example, direct positive feedback loops of C/EBP and PU.1 are crucial for switch-like behavior and are required for the stability of the granulocyte and monocyte phenotypes, respectively. Additional positive feedbacks loops exist within the mutually antagonistic architecture of the network. As C/EBP can antagonize PU.1 through Gfi-1, PU.1 forms two positive feedback loops by inhibiting C/EBP through IRF8 and by inhibiting Gfi-1 through Egr-2. These positive feedback loops are crucial to the stability of the monocytic phenotype. In contrast, C/EBP forms a positive feedback loop by activating Gfi-1, which in turn prevents PU.1 from upregulating IRF8 and inhibiting C/EBP. Thus C/EBP can exist at high concentrations by suppressing PU.1 through this positive feedback loop. Furthermore, Gfi-1 has a positive feedback mechanism by inhibiting PU.1 and Egr-2, which in turn would inhibit Gfi-1. These feedback loops are critical to the irreversibility of the granulocyte phenotype. More positive feedback mechanisms exist between receptors and transcription factors. For example, activated M-CSFR stimulates PU.1 and PU.1 stimulates the expression of M-CSFR. These types of positive feedback loops make the system more responsive to cytokine stimuli. Finally, C/EBP forms a negative feedback loop by activating PU.1 which in turn activates IRF8. This negative feedback loop is necessary for GM-CSF induced monopoiesis, as C/EBP must be suppressed, but only after it has activated PU.1.

Feedforward loops also make significant contributions to the behavior of the system. For instance, GM-CSFR activates C/EBP both directly and indirectly (by inhibiting IRF8), thus forming a coherent-feedforward loop. The inhibition of IRF8 by GM-CSF is important for GM-CSF induced differentiation of GMPs into M-MDSCs (analysis not shown). Another example is the incoherent-feedforward loop by which C/EBP activates PU.1 directly and inhibits PU.1 indirectly through Gfi-1. This incoherent-feedforward loop is crucial to the concentration dependent nature of GM-CSF induced differentiation, as we discussed previously.

### Limitations of model

While our model makes several intriguing predictions, we acknowledge that the model neglects many genes and proteins that play important roles in hematopoiesis. Therefore, in interpreting our models results, we must be aware of its limitations. We designed the model specifically to capture the initial decision-making stages of GMP differentiation, rather than the terminal stages of granulopoiesis and monopoiesis. We propose that transcription factors downstream of our network will play large roles in the maturation of granulocyte progenitor and monocyte cells, but only subtly effect the initial dynamics of lineage commitment.

Additionally, our model is limited to qualitative predictions. Although experiments often report quantitative measurements, it is impossible to compare these quantitative experimental results with our simulations for a variety of reasons. First of all, our calculations are made in dimensionless units, and the “real life” equivalent of 1 unit of M-CSF is not necessarily equivalent to 1 unit of G-CSF. Secondly, laboratory experiments typically utilize cytokine-enriched serum, with undefined serum components apparently necessary for cell survival and growth. These serum components are not accounted for in our model and may drastically impact how cells differentiate (87). Furthermore, cell-to-cell signaling, unaccounted in our model, may impact differentiation dynamics in experimental cultures. Finally, and perhaps the most important limitation of all, our model does not consider the impact of cytokine signaling on cellular responses such as proliferation and apoptosis. These responses may drastically impact the ratios of differentiated cells in experimental cultures. For example, while granulopoiesis may be favored under some conditions, rapid monocyte proliferation may skew experimental results in favor of a larger monocyte fraction.

### Summary

We have presented a novel model of GMP cell differentiation and explored the molecular control system's dynamics to provide insight into experimental observations and to make new predictions. We investigated the concentration-dependent nature of GM-CSF-induced differentiation, and proposed a mechanism that can explain its mysterious behavior. We explored the dynamics of CSF signaling crosstalk and found that, while G-CSF may encourage monopoiesis under some conditions, it is likely that M-CSF always has an inhibitory effect on granulopoiesis. Furthermore, our model demonstrates how both GMP cells and monocytes may differentiate into M-MDSCs, providing new insight into how this bizarre phenotype fits into classical GMP cell differentiation. We found that G-CSF, M-CSF, and GM-CSF may all favor M-MDSC development under different conditions. Moreover, we proposed that, among the CSFs, GM-CSF is the most potent inducer of this phenotype.

As for any “model” of a cellular control system, our model has limitations and potential sources of inaccuracy. For example, our model is not suitable for making quantitative predictions or capturing terminal states of GMP differentiation. Nonetheless, we are confident that our results have utility, as the dynamic processes captured by our model align with numerous experimental observations. Therefore, we welcome experimental evaluation of any of the qualitative predictions we have made.

## Author contributions

JT, BW, and LL were all involved in the project's conceptual development. BW designed and ran all computational simulations. BW developed all figures and wrote the first draft of the manuscript. All authors contributed to revising this draft into final form.

### Conflict of interest statement

The authors declare that the research was conducted in the absence of any commercial or financial relationships that could be construed as a potential conflict of interest.
