# Supplementary material for: Mathematical Analysis of Cytokine-Induced Differentiation of Granulocyte-Monocyte Progenitor Cells
Source: Front Immunol. 2018 Sep 18;9:2048. doi: 10.3389/fimmu.2018.02048 (PMC6153365; doi:10.3389/fimmu.2018.02048)
Supplement: Supplementary file 1 [file Presentation_1.PDF]

Supplementary Material

Mathematical Analysis of Cytokine-Induced Differentiation of Granulocyte-Monocyte Progenitor Cells

Bronson R. Weston, Liwu Li, and John J. Tyson*

*** Correspondence:** John J. Tyson: tyson@vt.edu

Model Tuning:

In consideration of the lack of time course data for protein concentrations during GMP differentiation, we went about tuning our model to match behavioral characteristics of GMP differentiation. We first defined the concentration profiles we would expect from these myeloid cells relative to each other (outlined below).

Cell Type	Higher Expression of:	Lower Expression of:
GMP	N/A	C/EBP, PU.1, IRF8. Egr-2, Gfi-1, GM-CSFR, M-CSFR, G-CSFR
Granulocyte	C/EBP, Gfi-1, G-CSFR	PU.1, Egr-2, IRF8, M-CSFR
Monocyte	PU.1, IRF8, Egr-2, M-CSFR, GM-CSFR	C/EBP, Gfi-1, G-CSFR
M-MDSC	C/EBP, PU.1, Egr-2, IRF8, M-CSFR, GM-CSFR	Gfi-1, G-CSFR

Next we defined the following characteristics we would expect under different signaling conditions:

- The GMP, granulocyte and monocyte states should all be stable under conditions with no stimuli. We would expect that granulocytes and monocytes, upon migrating out of areas of high CSF concentrations would remain stable and GMP cells should be reliant on the CSFs for differentiation.
- G-CSF should induce granulopoiesis and M-CSF should induce monopoiesis.
- GM-CSF should favor monopoeisis at lower concentrations and granulopoiesis at higher concentrations.
- G-CSF and M-CSF, when paired, should yield a heterogeneous population of monocytes and granulocytes.
- GM-CSF alone should produce a heterogeneous population of monocytes and granulocytes.
- Monocytes should not be able to convert back into GMPs or granulocytes regardless of cytokine exposure.

- Granulocytes should not be able to convert back into GMPs or monocytes regardless of cytokine exposure.
- GM-CSFR should be higher in the monocyte lineage than the granulocyte lineage.
- The M-MDSC state should be inducible by GM-CSF.

Next we chose weights that would result in the behaviors we defined. This is a lengthy and tedious process, and some weights are more relevant to certain behaviors than others. We will address a few of the weighting parameters, and their impact on the model's dynamics. First, the concentration dependent response of GMP cells to GM-CSF relies on a stronger interaction between C/EBP and Gfi-1 ($\omega_{Gfi1,C/EBP}$) than between C/EBP and PU.1 ($\omega_{PU.1,C/EBP}$). PU.1's auto-regulation gives it an advantage over Gfi-1 when C/EBP increases slowly, but if C/EBP increases faster the higher weight of its interaction with C/EBP gives it the upper hand. If the ratios between these weights were much smaller or much larger, GM-CSF would always favor one lineage over the other rather than exhibiting bi-functional behavior. Additionally, GM-CSFR is upregulated more heavily by PU.1 ($\omega_{GMCSFR,PU.1}$) than by C/EBP ($\omega_{GMCSFR,C/EBP}$). As a consequence, GM-CSFR is expressed higher in our simulated monocyte than in the granulocyte progenitor. Furthermore, the higher expression of GM-CSFR in monocytes makes them more sensitive to GM-CSF stimulation, and higher levels of GM-CSFR can shift the monocyte phenotype into an M-MDSC state. Receptor time scales (ρ_R) were set slower to transcription factor times scales (ρ_{TF}), which allows for a larger range of values for GM-CSF induced monopoiesis as it allows PU.1 more time to self-activate. Furthermore, we justify this distinction as transcription factors are often functional immediately after synthesis, while receptors must diffuse to the periphery of the cell and through the cell membrane, assemble with other subunits and with cytokines before a signal can be transmitted, and then the signal must propagate through multiple proteins for the signal itself to reach its downstream target.

Model Predictions:

- A combination of positive and negative interactions between C/EBP and PU.1 is responsible for the concentration dependent response of GMPs to GM-CSF.
- In the GMP state, Gfi-1 is more responsive to sudden shifts in C/EBP concentration than PU.1.
- GM-CSFR expression changes significantly after the cell has committed to one lineage in both granulopoiesis and monopoiesis. This suggests an alternative function for GMCSFR than lineage commitment.
- Low levels of G-CSF can push cells towards monopoiesis at M-CSF and GM-CSF concentrations too weak to stimulate differentiation alone.
- M-CSF antagonizes G-CSF induced granulopoiesis.
- GM-CSF induced monopoiesis exhibits a different concentration profile over time than M-CSF induced monopoiesis. In particular C/EBP and Gfi-1 spike in early stages of GM-CSF induced monopoiesis, and remain low in M-CSF induced monopoiesis.
- GM-CSF signal can increase the C/EBP concentration in monocytes.
- GM-CSF induced monopoiesis is likely quicker than M-CSF induced monopoiesis.
- GM-CSF induced granulopoiesis has a larger spike in PU.1 and IRF8 than G-CSF induced granulopoiesis. This is likely due to a slightly quicker rise in the concentration of Gfi-1 in G-CSF induced granulopoiesis.
- High expression of GM-CSFR in the monocyte lineage contributes to the sensitivity of GM-CSF induced M-MDSC differentiation.
- Gfi-1 is suppressed in the M-MDSC phenotype.
- The majority of C/EBP is bound to IRF8 in the monocyte lineage; whereas in M-MDSCs about half of C/EBP is unbound.
- M-MDSC differentiation resembles a monocyte in concentration profile before C/EBP is heavily upregulated.
- The M-MDSC state requires stimulation from cytokines other than M-CSF and G-CSF to remain stable.
- G-CSF can push monocytes into an M-MDSC phenotype in the presence of suitable concentrations of GM-CSF.
- M-CSF paired with GM-CSF makes for a stronger inducer of M-MDSCs than either cytokine alone.
- In M-MDSCs, G-CSFR is expressed at a level intermediate between a monocyte and a granulocyte.

Figures:

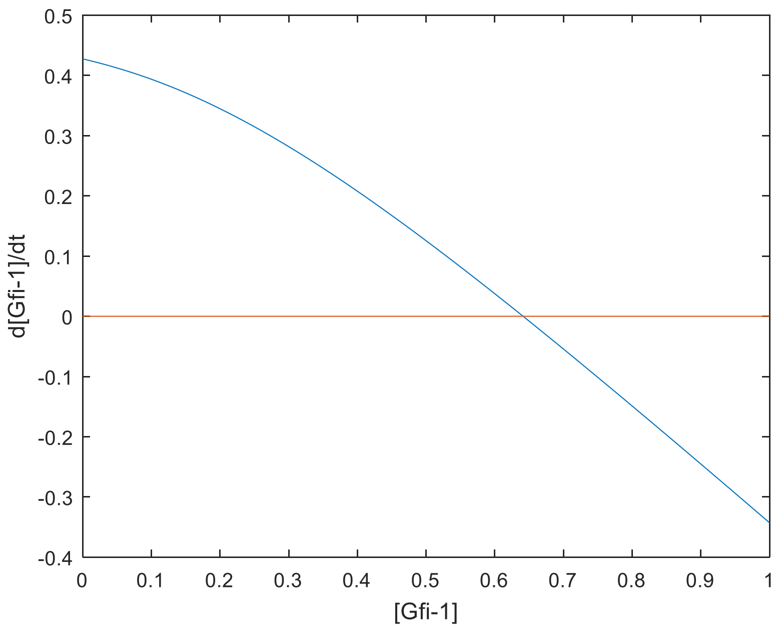

Figure SM1. An iterative search for the Gfi-1 solution. We search over a range of [Gfi1] values ([Gfi1] ∈ [0,1]) to find the steady state solution of [Gfi1], where the rate of change of [Gfi1] (blue curve) is equal to zero. The point where the blue curve intersects the red line is the location of the solution. When an intersection is found, we “zoom in” to find the solution by repeating the iterative search process with smaller intervals around the intersection until we find a solution that is within our tolerance specifications (10^{-9}). In this simulation, all CSF values = 0, [PU.1]=0.3, and $[C/EBP]_F$=0.6.

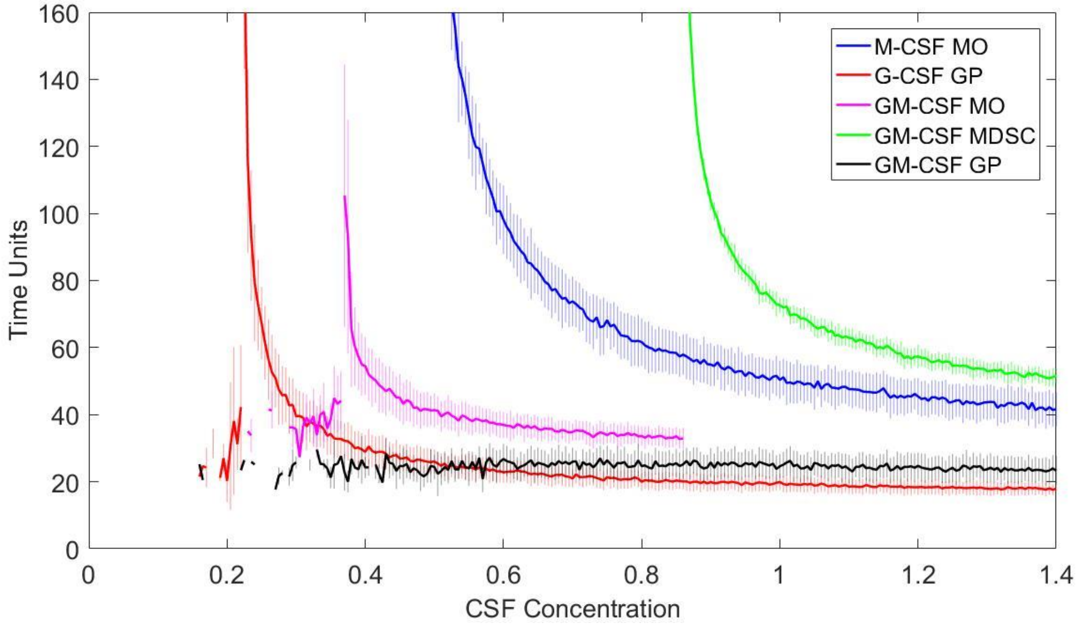

Figure SM2. Differentiation times under different cytokine conditions. Thick lines indicate the average time it takes a population of 100 cells to differentiate under the given cytokine concentration. Vertical lines represent the standard deviation of these differentiation times. The time it takes a GMP cell to differentiate decreases as the concentration of its stimulus increases, reaching a plateau at high CSF concentration. Results indicate that GM-CSF induced monopoiesis (magenta line) is quicker than M-CSF induced monopoiesis (blue line). Naturally, differentiation into the M-MDSC state (green line) takes substantially longer than monopoiesis, because the cell must first pass through a monocyte-like state before reaching the M-MDSC steady state. GM-CSF and G-CSF induced granulopoiesis have similar differentiation times. The asymptotic ascending differentiation times appear when the naïve state becomes unstable. The cells that differentiate at concentrations lower than this point are heavily primed to differentiate into the GP or MO phenotypes and thus will differentiate more quickly than the majority of cells.

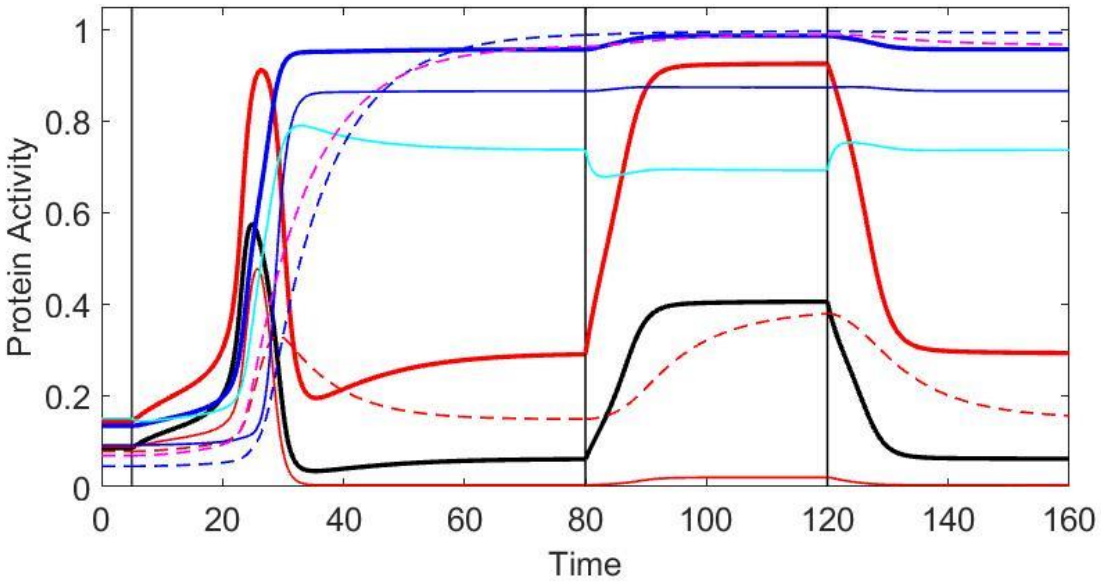

Figure SM3. GM-CSF can induce a monocyte to morph into an M-MDSC phenotype in a reversible process. A GMP cell differentiates into a monocyte when induced by lower concentrations of GM-CSF. However, when the concentration is increased further, the monocyte state destabilizes and the cell settles into the M-MDSC state. Furthermore, when the GM-CSF concentration is decreased again, the M-MDSC state is destabilized and reverts back to a monocyte-like phenotype. [GM-CSF] = 0 when $t < 5$. [GM-CSF] = 0.6 when $5 < t < 80$ and $t > 120$. [GM-CSF] = 1.2 when $80 < t < 120$. Thick red line = $[C/EBP]_T$, black line = $[C/EBP]_F$, thick blue line = [PU.1], thin red line = [Gfi1], thin blue line = [Egr2], cyan line = $[IRF8]_T$, dashed red line = $[G\text{-}CSFR]_T$, dashed blue line = $[M\text{-}CSFR]_T$, dashed magenta line = $[GM\text{-}CSFR]_T$.

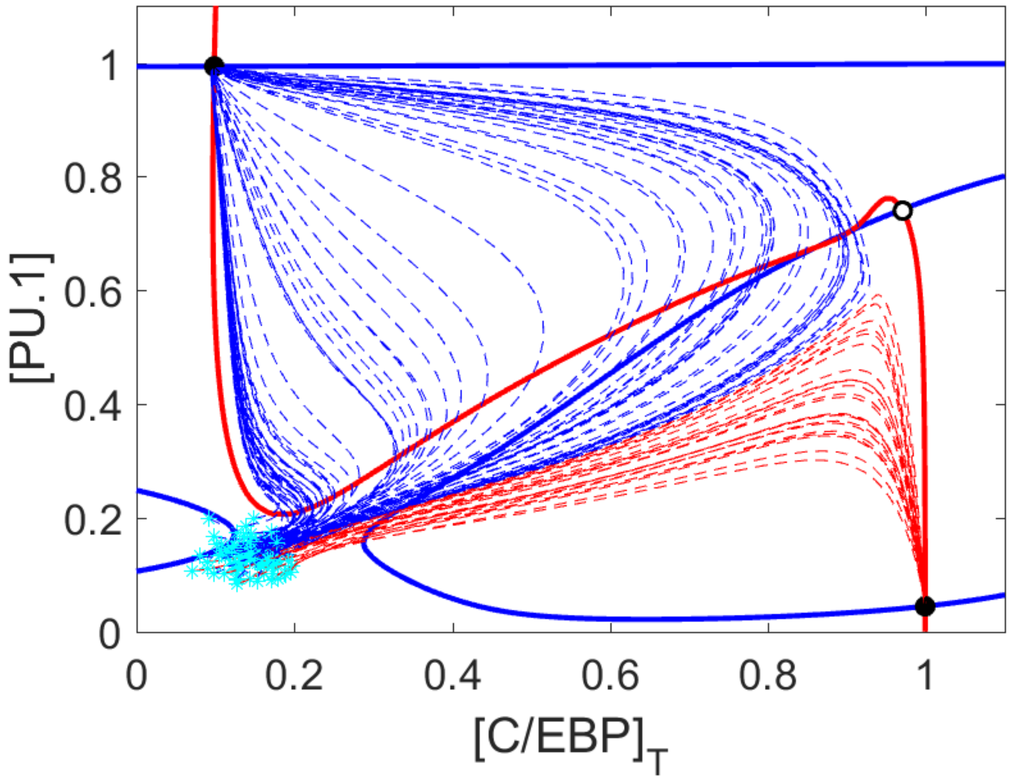

Figure SM4. Phase plane and cell trajectories describe the system under M-CSF and G-CSF stimulation. Thick, solid blue and red lines illustrate the PU.1 and C/EBP nullclines respectively. Cyan asterisks represent stochastically generated cellular initial conditions. Thin, red dashed lines represent the cell trajectory during granulopoiesis, while thin, blue dashed lines represent the cellular trajectories during monopoiesis. M-CSF and G-CSF can be mixed to get a heterogeneous population of both granulocytes and monocytes. As only the monocyte and granulocyte states are stable, M-MDSCs are not acquired under these conditions. [M-CSF] = 1 and [G-CSF] = 1 in this simulation.

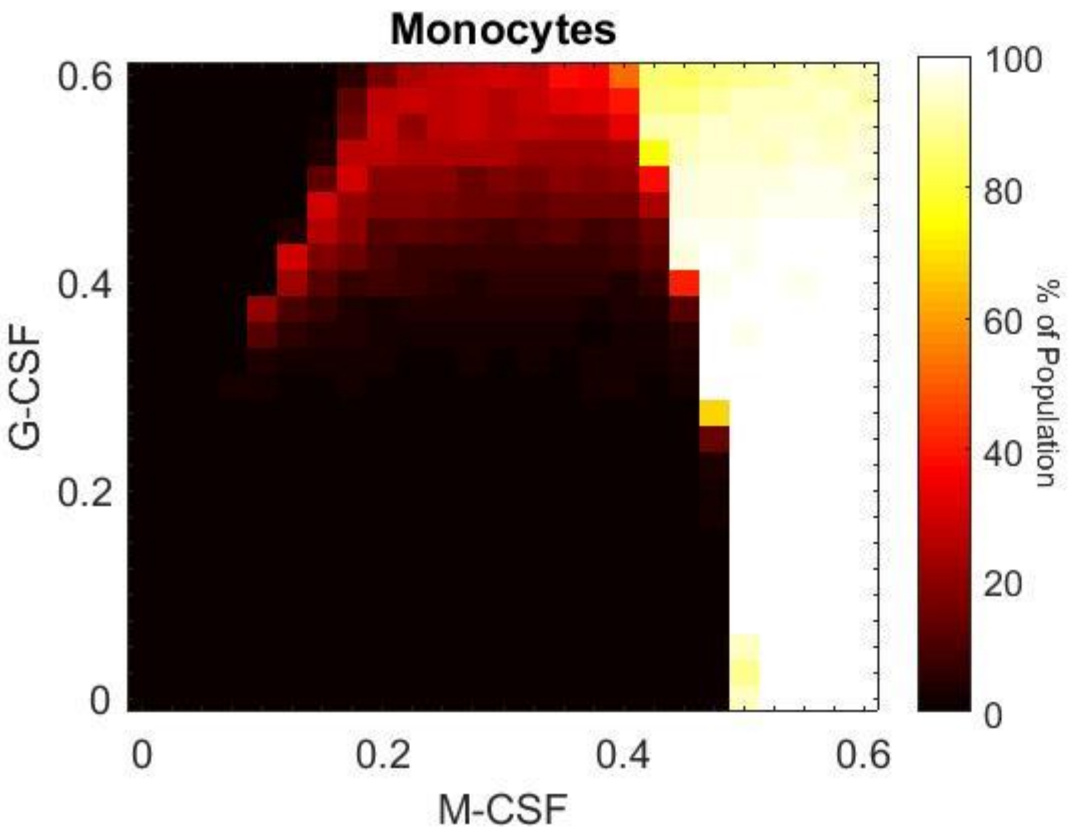

Figure SM5. Heat map of the population fraction that differentiates into monocytes under M-CSF and G-CSF stimulation. Each cytokine combination is simulated with 500 stochastically generated cells. The color gradient on the right correlates to fraction of cells that differentiate into monocytes. We find G-CSF can lower the required dose of M-CSF to induce monopoiesis. Thus, G-CSF can assist in monopoiesis under some conditions.

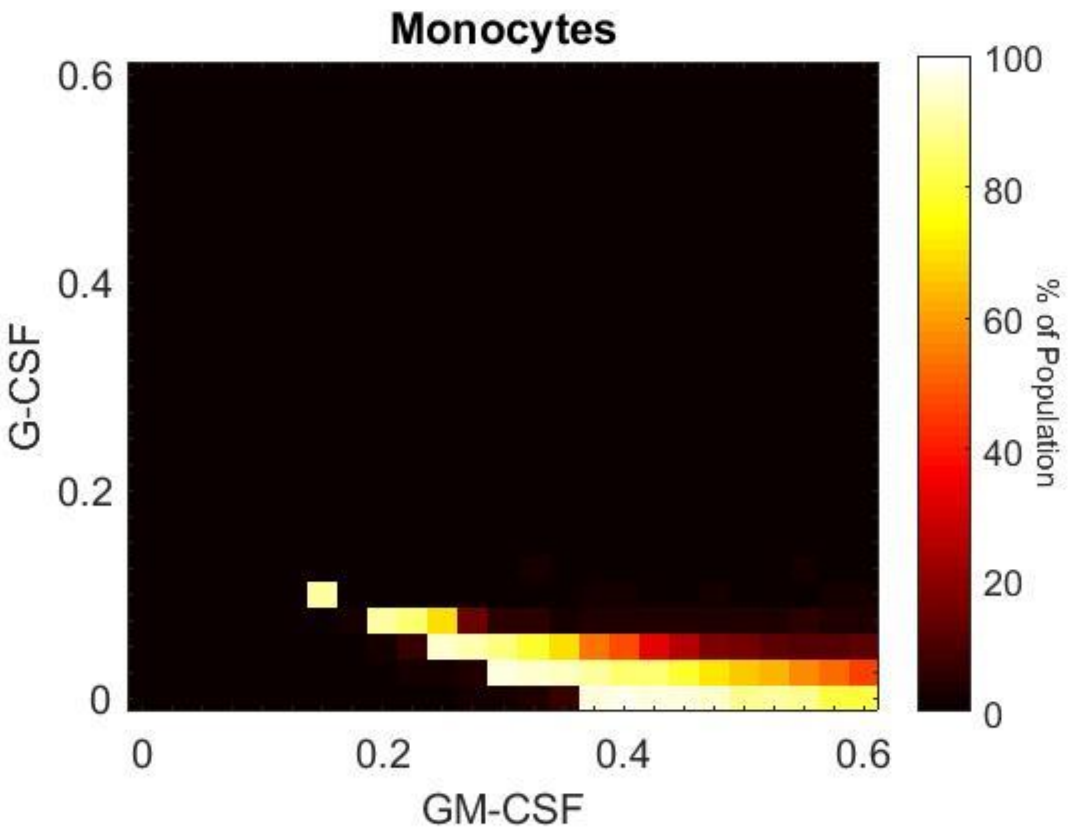

Figure SM6. Heat map of the population fraction that differentiates into monocytes under GM-CSF and G-CSF stimulation. Each cytokine combination is simulated with 500 stochastically generated cells. The color gradient on the right correlates to fraction of cells that differentiate into monocytes. We find G-CSF can lower the required dose of GM-CSF to induce monopoiesis. Thus, G-CSF can assist in monopoiesis under some conditions. However, when G-CSF is increased further, it arrests monopoiesis in favor of granulopoiesis.

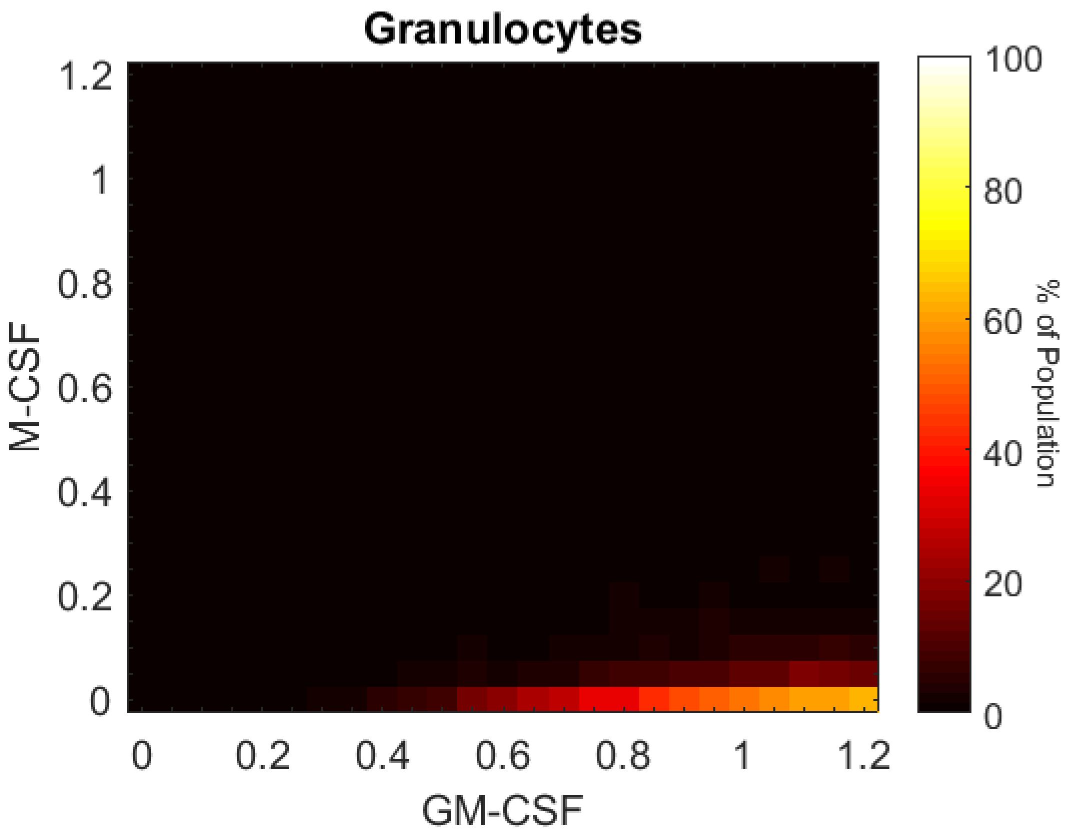

Figure SM7. Heat map of the population fraction that differentiates into granulocytes under GM-CSF and M-CSF stimulation. Each cytokine combination is simulated with 500 stochastically generated cells. The color gradient on the right correlates to fraction of cells that differentiate into granulocyte progenitors. We find that very low doses of M-CSF are required to arrest GM-CSF induced granulopoiesis.

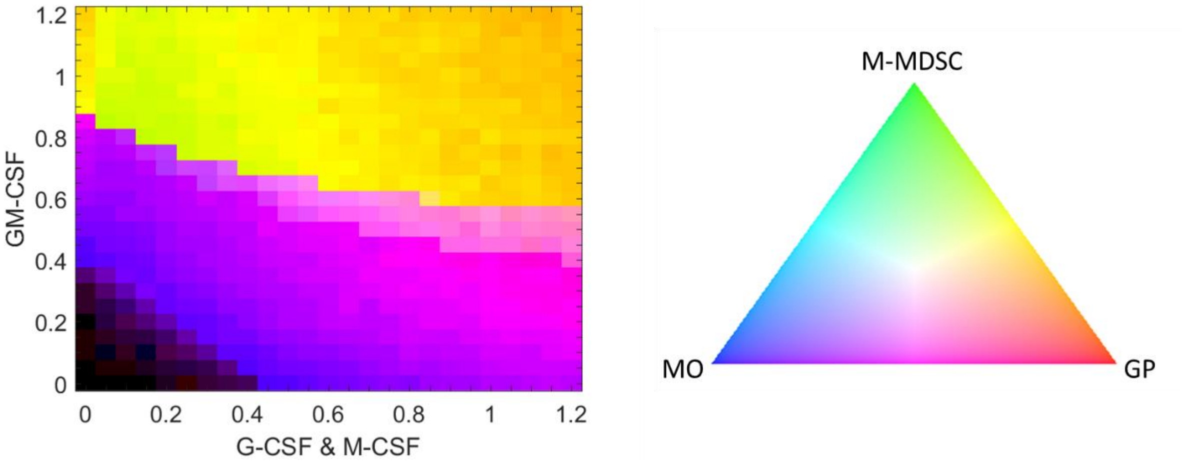

Figure SM8. Heatmap of GMP cell differentiation ratios when GM-CSF is paired with equal stimulation of G-CSF and M-CSF. Each cytokine combination is simulated with 500 stochastically generated cells. The color gradient triangle on the right correlates to population composition. Populations containing all M-MDSCs, granulocyte progenitors (GP), or monocytes (MO) are represented by green, red and blue, respectively. Black represents populations of undifferentiated cells. We find that as GM-CSF increases, the population ratio switches from being a blend of monocytes and granulocytes, to being composed of M-MDSCs and granulocytes. All three cell types are induced simultaneously under some conditions. As GM-CSF increases further, the ratio of granulocytes to M-MDSCs shifts in favor of granulocytes.

Table SM1. Equations

Differential Equations

1	$\frac{d[\mathrm{PU.1}]}{dt} = \rho_{\mathrm{TF}}\left(\frac{1}{1+e^{-\sigma W_{\mathrm{PU.1}}}} - [\mathrm{PU.1}]\right)$
2	$\frac{d[\mathrm{C/EBP}]_{\mathrm{T}}}{dt} = \rho_{\mathrm{TF}}\left(\frac{1}{1+e^{-\sigma W_{\mathrm{C/EBP}}}} - [\mathrm{C/EBP}]_{\mathrm{T}}\right)$
3	$\frac{d[\mathrm{Gfi1}]}{dt} = \rho_{\mathrm{TF}}\left(\frac{1}{1+e^{-\sigma W_{\mathrm{Gfi1}}}} - [\mathrm{Gfi1}]\right)$
4	$\frac{d[\mathrm{Egr2}]}{dt} = \rho_{\mathrm{TF}}\left(\frac{1}{1+e^{-\sigma W_{\mathrm{Egr2}}}} - [\mathrm{Egr2}]\right)$
5	$\frac{d[\mathrm{IRF8}]_{\mathrm{T}}}{dt} = \rho_{\mathrm{TF}}\left(\frac{1}{1+e^{-\sigma W_{\mathrm{IRF8}}}} - [\mathrm{IRF8}]_{\mathrm{T}}\right)$
6	$\frac{d[\mathrm{GMCSFR}]_{\mathrm{T}}}{dt} = \rho_{\mathrm{R}}\left(\frac{1}{1+e^{-\sigma W_{\mathrm{GMCSFR}}}} - [\mathrm{GMCSFR}]_{\mathrm{T}}\right)$
7	$\frac{d[\mathrm{GCSFR}]_{\mathrm{T}}}{dt} = \rho_{\mathrm{R}}\left(\frac{1}{1+e^{-\sigma W_{\mathrm{GCSFR}}}} - [\mathrm{GCSFR}]_{\mathrm{T}}\right)$
8	$\frac{d[\mathrm{MCSFR}]_{\mathrm{T}}}{dt} = \rho_{\mathrm{R}}\left(\frac{1}{1+e^{-\sigma W_{\mathrm{MCSFR}}}} - [\mathrm{MCSFR}]_{\mathrm{T}}\right)$

***W* equations**

9	$W_{\mathrm{PU.1}} = \omega^{\mathrm{o}}_{\mathrm{PU.1}} + \omega_{\mathrm{PU.1,PU.1}}[\mathrm{PU.1}] + \omega_{\mathrm{PU.1,Gfi1}}[\mathrm{Gfi1}] + \omega_{\mathrm{PU.1,C/EBP}}[\mathrm{C/EBP}]_{\mathrm{F}} + \omega_{\mathrm{PU.1,MCSFR}}[\mathrm{MCSFR{:}\ MCSF}]$
10	$W_{\mathrm{C/EBP}} = \omega^{\mathrm{o}}_{\mathrm{C/EBP}} + \omega_{\mathrm{C/EBP,C/EBP}}[\mathrm{C/EBP}]_{\mathrm{F}} + \omega_{\mathrm{C/EBP,GMCSFR}}[\mathrm{GMCSFR{:}\ GMCSF}] + \omega_{\mathrm{C/EBP,GCSFR}}[\mathrm{GCSFR{:}\ GCSF}]$
11	$W_{\mathrm{Gfi1}} = \omega^{\mathrm{o}}_{\mathrm{Gfi1}} + \omega_{\mathrm{Gfi1,C/EBP}}[\mathrm{C/EBP}]_{\mathrm{F}} + \omega_{\mathrm{Gfi1,Egr2}}[\mathrm{Egr2}] + \omega_{\mathrm{Gfi1,GCSFR}}[\mathrm{GCSFR{:}\ GCSF}]$
12	$\mathrm{W}_{\mathrm{Egr2}} = \omega^{\mathrm{o}}_{\mathrm{Egr2}} + \omega_{\mathrm{Egr2,PU.1}}[\mathrm{PU.1}] + \omega_{\mathrm{Egr2,Gfi1}}[\mathrm{Gfi1}]$
13	$W_{\mathrm{IRF8}} = \omega^{\mathrm{o}}_{\mathrm{IRF8}} + \omega_{\mathrm{IRF8,PU.1}}[\mathrm{PU.1}] + \omega_{\mathrm{IRF8,GMCSFR}}[\mathrm{GMCSFR{:}\ GMCSF}] + \omega_{\mathrm{IRF8,GCSFR}}[\mathrm{GCSFR{:}\ GCSF}]$
14	$W_{\mathrm{GMCSFR}} = \omega^{\mathrm{o}}_{\mathrm{GMCSFR}} + \omega_{\mathrm{GMCSFR,PU.1}}[\mathrm{PU.1}] + \omega_{\mathrm{GMCSFR,C/EBP}}[\mathrm{C/EBP}]_{\mathrm{F}}$
15	$W_{\mathrm{GCSFR}} = \omega^{\mathrm{o}}_{\mathrm{GMCSFR}} + \omega_{\mathrm{GCSFR,PU.1}}[\mathrm{PU.1}] + \omega_{\mathrm{GCSFR,C/EBP}}[\mathrm{C/EBP}]_{\mathrm{F}} + \omega_{\mathrm{GCSFR,Gfi1}}[\mathrm{Gfi1}]$

16 $$W_{\mathrm{MCSFR}} = \omega^{\mathrm{o}}_{\mathrm{MCSFR}} + \omega_{\mathrm{MCSFR,PU.1}}[\mathrm{PU.1}] + \omega_{\mathrm{MCSFR,C/EBP}}[\mathrm{C/EBP}]_{\mathrm{F}} + \omega_{\mathrm{MCSFR,Gfi1}}[\mathrm{Gfi1}] + \omega_{\mathrm{MCSFR,Egr2}}[\mathrm{Egr2}]$$

Protein Binding Equations

17 $$[\mathrm{GMCSFR:GMCSF}] = \frac{[\mathrm{GMCSF}][\mathrm{GMCSFR}]_{\mathrm{T}}}{[\mathrm{GMCSF}] + K_{\mathrm{d,GMCSFR}}}$$

18 $$[\mathrm{MCSFR:MCSF}] = \frac{[\mathrm{MCSF}][\mathrm{MCSFR}]_{\mathrm{T}}}{[\mathrm{MCSF}] + K_{\mathrm{d,MCSFR}}}$$

19 $$[\mathrm{GCSFR:GCSF}] = \frac{[\mathrm{GCSF}][\mathrm{GCSFR}]_{\mathrm{T}}}{[\mathrm{GCSF}] + K_{\mathrm{d,GCSFR}}}$$

20 $$[\mathrm{C/EBP}]_{\mathrm{F}} = \frac{1}{2}\left(-b + \sqrt{b^2 + \frac{4[\mathrm{C/EBP}]_{\mathrm{T}}}{K_{\mathrm{eq}}}}\right)$$

21 $$b = [\mathrm{IRF8}]_{\mathrm{T}} + \frac{1}{K_{\mathrm{eq}}} - [\mathrm{C/EBP}]_{\mathrm{T}}$$

Table SM2. Basal parameter values. Cytokine concentrations vary from one simulation to another, as specified in the figure legends. The half-life of transcription factors is ~$(0.7/\rho_{\mathrm{TF}})\cdot(60\ \mathrm{min})$ = 42 min, and for receptors ~420 min.

Targeted Protein	Parameter	Value
**General terms**	σ	3.2
	ρ_{TF}	1
	ρ_{R}	0.1
	[GM-CSF]	adjustable
	[G-CSF]	adjustable
	[M-CSF]	adjustable
**C/EBP**	$\omega^{\mathrm{o}}_{\mathrm{C/EBP}}$	- 0.79
	$\omega_{\mathrm{C/EBP,C/EBP}}$	2.71
	$\omega_{\mathrm{C/EBP,GMCSFR}}$	0.75
	$\omega_{\mathrm{C/EBP,GCSFR}}$	0.85
	K_{eq}	7.5
**PU.1**	$\omega^{\mathrm{o}}_{\mathrm{PU.1}}$	- 0.8
	$\omega_{\mathrm{PU.1,PU.1}}$	1.79
	$\omega_{\mathrm{PU.1,Gfi1}}$	- 1.22
	$\omega_{\mathrm{PU.1,C/EBP}}$	0.99
	$\omega_{\mathrm{PU.1,MCSFR}}$	0.85
**Gfi-1**	$\omega^{\mathrm{o}}_{\mathrm{Gfi1}}$	- 0.75
	$\omega_{\mathrm{Gfi1,C/EBP}}$	1.6
	$\omega_{\mathrm{Gfi1,Egr2}}$	- 1.27
	$\omega_{\mathrm{Gfi1,GCSFR}}$	0.75
**IRF8**	$\omega^{\mathrm{o}}_{\mathrm{IRF8}}$	- 0.73
	$\omega_{\mathrm{IRF8,PU.1}}$	1.4
	$\omega_{\mathrm{IRF8,GMCSFR}}$	- 0.62
	$\omega_{\mathrm{IRF8,GCSFR}}$	- 0.62
**Egr2**	$\omega^{\mathrm{o}}_{\mathrm{Egr2}}$	- 0.8
	$\omega_{\mathrm{Egr2,PU.1}}$	1.45
	$\omega_{\mathrm{Egr2,Gfi1}}$	- 1.27
**GM-CSFR**	$\omega^{\mathrm{o}}_{\mathrm{GMCSFR}}$	- 1.2
	$\omega_{\mathrm{GMCSFR,PU.1}}$	2.3
	$\omega_{\mathrm{GMCSFR,C/EBP}}$	0.9
	$K_{\mathrm{d,GMCSFR}}$	0.65
**G-CSFR**	$\omega^{\mathrm{o}}_{\mathrm{GMCSFR}}$	- 1.0
	$\omega_{\mathrm{GCSFR,PU.1}}$	0.4
	$\omega_{\mathrm{GCSFR,C/EBP}}$	1.1
	$\omega_{\mathrm{GCSFR,Gfi1}}$	0.9
	$K_{\mathrm{d,GCSFR}}$	0.9

**M-CSFR**	$\omega^{o}_{\mathrm{MCSFR}}$	- 1.25
	$\omega_{\mathrm{MCSFR,PU.1}}$	2
	$\omega_{\mathrm{MCSFR,C/EBP}}$	0.5
	$\omega_{\mathrm{MCSFR,Gfi1}}$	- 1.2
	$\omega_{\mathrm{MCSFR,Egr2}}$	1
	$K_{\mathrm{d,MCSFR}}$	0.45

Table SM3. Initial Conditions. All time course simulations, except stochastically generated cells, were run with the following initial conditions:

$[\mathrm{C/EBP}]_T$ = 0.1424	[PU.1] = 0.1331	[Gfi1] = 0.08803	$[\mathrm{GMCSFR}]_T$ = 0.06807
$[\mathrm{IRF8}]_T$ = 0.1494	[Egr2] = 0.09112	$[\mathrm{GCSFR}]_T$ = 0.07737	$[\mathrm{MCSFR}]_T$ = 0.04482
